# Harnessing genetic variation for grain quality from diverse wheat landraces

**DOI:** 10.1111/nph.71628

**Published:** 2026-10-09

**Authors:** Luqman B. Safdar, M. John Foulkes, Alix Cornish, Elizabeth D. Goodman, Andrew B. Riche, David Steele, Charlie Philp, Shiva Rahimitanha, Martin Whitworth, Iain R. Searle, Matt Kerton, Malcolm J. Hawkesford, Simon Griffiths, Rahul A. Bhosale, Scott A. Boden, Ian D. Fisk

**Affiliations:** ^1^ School of Biosciences University of Nottingham Sutton Bonington LE12 5RD UK; ^2^ School of Agriculture, Food and Wine Adelaide University Glen Osmond SA 5064 Australia; ^3^ John Innes Centre Norwich Research Park Norwich NR4 7UH UK; ^4^ Campden BRI Chipping Campden GL55 6LD UK; ^5^ Rothamsted Research Harpenden AL5 2JQ UK; ^6^ School of Biological Sciences Adelaide University Adelaide SA 5005 Australia; ^7^ DSV UK Ltd Top Dawkins Barn Wardington OX17 1FE UK

**Keywords:** genome‐wide association study, grain protein content, grain quality, hyperspectral imaging, QTL mapping, *Triticum aestivum* L, wheat landraces

## Abstract

- The grain quality traits of protein content, grain weight and size are socially and economically important for wheat (*Triticum aestivum*) breeding. Modern wheat germplasm has limited genetic variation for vital traits, including grain quality, underscoring the need to investigate ancestral varieties/landraces for beneficial allelic diversity.
- Here, we develop a reliable and sensitive single‐seed hyperspectral imaging‐based phenotyping platform to quantify grain protein, weight and size in 827 diverse wheat landraces, achieving improved predictive accuracy (*R* ^2^ = 0.91) vs previous methods. We identified novel genetic variants (or loci) for grain protein content and its heterogeneity between individual grains in a genotype, and grain weight, and validated them through bi‐parental linkage mapping.
- Many loci we report are rare and absent from modern breeding germplasm. Beneficial haplotypes at these loci improved mean protein content and homogeneity without compromising grain weight; these haplotypes represent valuable genetic variation for agriculture. We identify a stable locus on chromosome 6A contributing to protein heterogeneity within single grains, suggesting homogeneity in protein content could be a selectable trait for breeding.
- Our findings demonstrate how integration of advanced phenotyping into breeding strategies can harness untapped genetic diversity for grain quality improvement in wheat, providing an opportunity to enhance grain nutritional value.

## Graphical abstract

Grain protein content is usually measured on bulk samples, hiding variation between individual grains. We used hyperspectral imaging to predict protein in *c*. 15 000 single grains from over 500 Watkins wheat landraces grown in two environments, resolving both mean protein content and grain‐to‐grain uniformity. Genome‐wide association mapping identified six protein loci, two grain weight loci and SGPH‐A1 on chromosome 6A controlling uniformity. Haplotypes at 6D raised protein without reducing grain weight, and haplotypes at 1A and 6A raised grain weight without diluting protein, offering routes to break the protein–yield trade‐off.

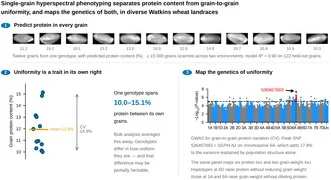

## Introduction

Grain protein content (GPC) in wheat is a nutritionally and economically important trait as it determines the rheological properties of dough and the grain's market value. Higher GPC is associated with better gluten development that is desirable for breadmaking (Hoseney, 1994; Shewry, 2019). Major factors affecting GPC variation are environment, nitrogen (N) availability, genotype by environment (G × E) interaction and genetic variation (Shewry, 2007). Improvement of GPC is often limited due to a trade‐off with grain yield; selection of genetic variants that improve GPC and its stability across environments must minimize this trade‐off. A well‐established approach for identifying beneficial genetic variation involves exploring diverse populations that incorporate large‐scale diversity (Bailey‐Serres *et al*., 2019). A key step is accurate phenotyping of large populations over several years and environments. Phenotyping for GPC through conventional methods (e.g. Kjeldahl or Dumas Combustion) among large populations can be challenging due to low throughput, high costs and intensive labour inputs. These methods are also destructive, limiting population‐level investigation of GPC in early generations. Hyperspectral imaging (HSI) has emerged as a phenotyping method suitable for analysing GPC (Sarić *et al*., 2022). The single‐grain phenotyping capabilities provided by HSI facilitate the investigation of GPC variation that can exist in a plot or within a cultivar (Delwiche, 1995; Delwiche, 1998; Tokatlidis *et al*., 2004; Caporaso *et al*., 2018; Ninou *et al*., 2022). Such intra‐cultivar trait heterogeneity is commonly associated with factors including cropping density, tiller cohorts, spikelet positional effect or soil properties (Herrera, 2009; Tokatlidis *et al*., 2011; March‐Salas *et al*., 2020). However, it remains unclear whether GPC heterogeneity is genetically regulated or influenced by known GPC alleles, limiting breeders' ability to develop cultivars with more uniform protein content.

HSI is a non‐destructive surface level technique that enables the investigation of the genetic architecture underlying GPC (Safdar *et al*., 2023). Uniform grains are preferred by millers because they allow for more efficient processing using consistent equipment settings. Additionally, increased homogeneity should produce fewer small grains, which may be lost during machine harvesting. HSI can also measure grain morphological parameters, such as length and area, enabling simultaneous assessment of GPC alleles and their effect on grain size. This capability impacts breeding because an increase in GPC through a dilution effect (smaller grains producing higher GPC) is not desirable. To date, efforts to predict grain weight (GW) using HSI have had limited success (Caporaso *et al*., 2018). Advancing HSI‐based models for GW would enable simultaneous investigation of the interplay between GPC and GW, improving analysis of grain quality across large diversity panels.

Genetics of GPC is a moderately established research area, with the first major quantitative trait locus (QTL), *Gpc‐B1* (*QGpc.nsdu.6Bb*), identified on chromosome 6B about three decades ago (Joppa *et al*., 1997). The effect of *Gpc‐B1* is underpinned by variant alleles for a gene encoding a NAC domain protein, *NAM‐B1*, which influences grain protein by altering post‐anthesis remobilization of N from leaves to spikes and accelerating senescence (Uauy *et al*., 2006). The *Gpc‐B1* functional allele improves GPC without penalizing yield in many cases, but its effect varies in different environments (Tabbita *et al*., 2017). Given that *Gpc‐B1* was contributed by a wild emmer wheat, other exotic wheat genotypes may harbour novel beneficial GPC alleles. Recently, the AE Watkins collection – 827 world‐wide wheat landraces – was sequenced at the whole genome level, revealing that modern wheats emerged from only two of the seven ancestral groups (AGs; Cheng *et al*., 2024). Thus, Watkins landraces are enriched with genetic diversity absent in modern wheats, offering opportunities to discover novel variants influencing grain quality traits.

By integrating HSI with quantitative genetics, we predicted GPC and GW in two field trials of the Watkins collection and identified novel QTL on chromosomes 1A, 6A, 6D and 7D, which harbour beneficial haplotypes for GPC and GW. Furthermore, we demonstrate that GPC heterogeneity is influenced by genetic variation, with a stable QTL on chromosome 6A detected in multiple environments.

## Materials and Methods

### Plant material

Three sample sets were obtained for the experiments, referred to as S1, S2 and S3. S1 comprised 124 genotypes of Watkins wheat (*Triticum aestivum* L.) landraces including 47 core lines, used to collect ground‐truth protein data from single grains for model development. Core lines are a set of 119 Watkins landraces capturing most of the collections' genetic diversity (Wingen *et al*., 2014). Seed was sourced from the John Innes Centre (JIC), curated from several field/glasshouse experiments. S2 comprised 689 genotypes of Watkins landraces, including 69 core lines grown in the field at JIC 2019‐20. S3 comprised 610 genotypes of Watkins landraces including 92 core lines grown in the field at Rothamsted Research (RRes; 2021‐22). The field experiments S2 and S3 used randomized complete block designs with three replicates. Twelve grains per genotype were selected from a single plot per grain trait (GPC, grain morphology) analysis. The Watkins collection comprises 827 landraces from 32 countries, and is maintained at JIC (https://www.seedstor.ac.uk; Wingen *et al*., 2014; Cheng *et al*., 2024). S1 was used for the development of a calibration model for predicting GPC from single grains. S2 and S3 were used to phenotype GPC, GPC heterogeneity, GW and morphological traits from two field trials for genetic analysis.

### Hyperspectral imaging

Hyperspectral images of single wheat grains were acquired using a pushbroom approach. The instrument was supplied by Gilden Photonics Ltd (Glasgow, UK) and includes a SWIR spectral camera (Specim Ltd, Oulu, Finland) containing a cooled 14‐bit 320 × 256 pixel HgCdTe detector and N25E spectrograph. This provides 256 spectral bands over wavelengths 990–2500 nm, with a spectral resolution of 6 nm. Single‐grain samples were imaged at a resolution of 0.109 mm pixel^−1^. The camera shutter was closed for 1 s at the end of each scan to collect the dark reference, and *c*. 100 frames were recorded per session from a white (polytetrafluoroethylene) PTFE reference material with *c*. 100% reflectance across the entire measured spectral range (white reference).

For S1 used for calibration, all 248 grains were scanned in both crease‐down and crease‐up orientations. For S2 and S3, for each sample, 12 single grains per scan were placed in a grid formation on a black high‐density polyethylene tray, with half positioned crease‐down and the other half crease‐up. The position of grain 1 was identified using a white triangle marker.

### Acquisition of average spectra

Hyperspectral images were analysed using *spectral* library in python 3.9.7. Spikes caused by bad pixels were removed using *median_filter* from *scipy.ndimage*. Data were calibrated by subtracting the dark reference and normalized by dividing by the white reference. Normalized reflectance images were converted into absorbance images (log1/R) to increase the contrast. Band 91–130 from the absorbance images were averaged and *threshold_otsu* from *skimage.filters* was used for creating a binary mask. Otsu's method is a well‐known thresholding technique that automatically determines the optimal threshold value from the image histogram. Regions of interest were selected using *regionprops* function from *skimage.measure*. Edges were eroded using *binary_erosion* from *scipy*.ndimage, and mean spectra were calculated for each grain.

### N determination

HSI reflectance data and GPC measured from N% determination on single wheat grains were used to develop a GPC prediction model. Single‐grain samples were tested for determination of N content by Dumas combustion using a LECO FP628 based on CCAT method no. 19 TES‐CM‐119 (non‐UKAS). N content of individual grain was multiplied by 5.7 to estimate GPC (Müller, 2017). HSI and Dumas analyses were performed at Campden BRI (Chipping Campden, UK).

### Calibration for grain protein content

Ground‐truth GPC data for 248 single grains (from S1) and reflectance measured at 239 spectral bands ranging from 999 to 2494 nm were entered into a partial least squares regression (PLSR) model for training. As HSI data were obtained from both crease‐up and crease‐down orientations of grains, 496 data points were available. Data for two genotypes were excluded due to abnormal measurements. Four PLSR models (named M1 to M4) were tested for the remaining 488 samples. Of these, 368 were used for training and 120 were separated into two test sets – one with 60 samples crease‐down and the second with 60 samples crease‐up. For M1, spectral data were normalized using Standard Normal Variate (SNV) and Savitzky–Golay 1st Derivative (SG‐1). Spectral data normalization was performed to remove multiplicative scattering effects and noise. For M2, data were normalized using SNV and SG‐2. For M3 and M4, spectral data from different orientations of a grain were averaged to produce one spectral profile per grain, leaving 184 samples in the training set. As before, normalization for M3 was performed by SNV and SG‐1, while SNV and SG‐2 were used for M4. All models were tested on the two test sets and that with the least difference in predicting GPC for different grain orientations was selected. After selection of optimal modelling parameters, all data were recombined for final model development. Training data were randomly partitioned into five equal‐sized subsets, of which four subsets were used as training data and a single subset was retained for validation. The cross‐validation was repeated five times with each subset used once as validation data and the coefficient of determination (*R* ^2^) and root mean square error (RMSE) were calculated for each run. All the functions used in modelling were employed from *scikit‐learn* library in python‐3.9.7 (Pedregosa *et al*., 2011).

From S2 and S3, 12 grains per genotype (6 crease‐up and 6 crease‐down) were scanned using HSI and their individual protein values were predicted using the PLSR model with best parameters. From the predicted protein values, outliers were removed using the Modified *Z*‐score method with a cut off value of 3.5. Descriptive statistics were calculated from the cleaned data including mean and coefficient of variation (%) between 12 grains in a genotype which were described as GPC and GPC heterogeneity, respectively. If a variable was not normally distributed, it was transformed using either of Square Root, Box‐Cox or Yeo‐Johnson transformations as per suitability. These statistical tests were performed in python‐3.9.7 using *numpy*, *pandas* and *scipy.stats* libraries (McKinney, 2010; Harris *et al*., 2020; Virtanen *et al*., 2020); plots were generated using *matplotlib* and *seaborn* libraries (Hunter, 2007; Waskom, 2021).

Broad‐sense heritability was determined as: $H^{2}=V_{g}/V_{p}$, where *V*_g_ represents genetic variance and *V*_p_ represents phenotypic variance. *V*p was partitioned into *V*_g_, *V*_ge_ (genotype × environment interaction variance) and *V*_e_ (environment variance including residuals). A mixed effect model was adopted to calculate variance components with ‘Year’ as fixed effect to estimate the significance of difference between years, ‘Genotype’ as random effect to estimate genetic variance while treating genotypes as random samples of a population and ‘Genotype *×* Environment’ as a random effect to estimate environmental variance. The model formula was: *Y*_*ijk*_ *= μ + G*_*i*_ *+ E*_*j*_ + (*G × E*)_*ij*_ + *ɛ*, where: *Y*_*ijk*_ is the GPC value for *k* ^th^ replicate of *i*‐th genotype in *j* ^th^ year; *μ* is the overall mean for GPC; *G*_*i*_ is the effect of *i* ^th^ genotype; *E*_*j*_ is the effect of *j* ^th^ year; (*G* × *E*)_*ij*_ is the interaction between *i* ^th^ genotype and *j* ^th^ year; *ɛ* is the residual error.

As data for single grains were collected from two field trials (JIC and RRes in different seasons) a high *G* × *E* interaction was expected. Therefore, a second model was also fitted ignoring the *G* × *E* interaction effect as: *Y*_*ijk*_ *= μ + G*_*i*_ *+ E*_*j*_ *+ ɛ* and *V*p was estimated as *V*_g_ + *V*_e_ where *V*_e_ represented environmental variance including the residuals. The models were fitted using *mixedlm* from *statsmodel.api*.

### Measurement of grain morphological features

Shape and size features including grain length (GL), breadth, area, roundness, perimeter, ellipticity, compactness and roughness were calculated using pixel information from the hyperspectral images. GL was calculated using pixel number multiplied by pixel size along the longitudinal axis of the grain, while width was calculated along the lateral axis.

### Calibration for grain weight

The measured GW of 1352 grains and morphological features were used to train a PLSR model for GW. Normalization was performed using the *StandardScaler* function from *sklearn.preprocessing*, which uses *z*‐score normalization to help centre the data around zero and scale it to have a SD of 1. Data were randomly split into a training set (80%) and a test set (20%) using *train_test_split*, as described for GPC. Model performances were evaluated based on *R* ^2^ and RMSE of prediction on the test set.

### Genome‐wide association analyses

Sequence data for Watkins landraces were available as part of the WatSeq Project under the Global Grain Genomics Research Program (Cheng *et al*., 2024). This included over 90 million single nucleotide polymorphisms (SNPs) in 827 Watkins landraces, already filtered for missingness, Hardy–Weinberg equilibrium and minor allele frequency (Cheng *et al*., 2024). The filtered SNP dataset was pruned using a two‐step linkage disequilibrium (LD) pruning approach in PLINK‐1.9. The panel structure from WatSeq that divided the Watkins collection into seven ancestral subgroups was used as covariates. To estimate the genetic relatedness between samples, kinship analysis was performed in GEMMA‐0.98.5 with parameters *gemma ‐bfile genotype ‐gk 1 ‐o kinship*. GWAS analysis was performed using a linear mixed model (LMM) with parameters *gemma ‐bfile genotype ‐c structure ‐k kinship ‐p phenotype ‐lmm 1 ‐miss 0.9 ‐o output*. Wald (with *‐lmm* 1) and Log Likelihood Ratio (with *‐lmm 2*) tests were both tested for one variable, and when no difference was observed, the Wald test was maintained due to computational efficiency. LMM tests were performed for the GPC and its heterogeneity in 2020, 2022 and the average data.

### Identification of candidate loci and beneficial haplotypes

All SNPs that were significant (*P* value 1e‐5; −log_10_ *P* ≥ 5) were investigated further. A 5 Mb sliding window was used for initial locus designation, supported by previous studies reporting LD extending up to 9 Mb in different bread wheat varieties (Brinton *et al*., 2020). Cultivated wheat varieties tend to have longer LD blocks due to selective breeding, compared to landraces which represent more genetic diversity hence shorter LD blocks (Hao *et al*., 2011). Any GPC loci that were significant in at least two of the three data (i.e. 2020, 2022 and average) were considered for further investigation; however, a modified approach was adopted for GPC heterogeneity.

To account for the high genotype × site‐season interaction expected and increase confidence in loci, a subset of genotypes was created by comparing the coefficient of variation (CV%) values for the genotypes in the two site‐seasons and excluding those showing most divergence across site‐seasons based on a 25% difference threshold. This resulted in a subset of 263/509 common genotypes across the 2 yr. Another GWAS was performed on these 263 samples and loci for these data that were common with the full dataset were investigated further.

Once candidate loci were selected, pairwise LD was estimated using Haploview‐4.2 to determine the intervals of LD blocks and identify haplotypes (Barrett *et al*., 2005). The haplotype effect on trait was analysed by Independent Samples *t*‐test using *ttest_ind* function from *scipy.stats* or ANOVA using *ols* followed by *pairwise_tukeyhsd* from *statsmodels* when more than two haplotype groups (Seabold & Perktold, 2010; Virtanen *et al*., 2020). QTL were defined based on LD block interval around the tag SNP (with the lowest *P* value); all LD blocks in which haplotypes influenced trait were kept in the QTL interval. The proportion of phenotypic variance explained (PVE %) by a QTL was calculated by multiple linear regression first using structure as the baseline model and subsequently adding SNPs to the model. To increase confidence in the prioritized GPC QTL, we investigated for haplotypes in the corresponding regions from the Watkins HapMap (published by Cheng *et al*. (2024)) and their effect on GPC‐related traits.

### Stability of haplotype effects across environments

Haplotype effect stability was assessed using accessions phenotyped in both environments. For each locus, haplotypes represented by at least five accessions were retained and effects expressed relative to the major haplotype, estimated separately in each environment and compared by Welch's *t*‐test. Haplotype × environment interaction was tested by regressing the within‐accession difference between environments (2022–2020) on haplotype; as this difference is taken within accessions, it cancels all accession‐level effects, including population structure, relatedness and any constant measurement offset. One interaction test was performed per locus block, and *P* values were Holm‐adjusted across all locus‐blocks tested within each trait. Because the two environments differ in both site and season, interaction cannot be partitioned into site and year components.

### Candidate gene identification

Three approaches were used to provide a holistic view of genes underlying prioritized loci, which included expression‐based, annotation‐based and Watkins‐unique variation‐based investigations that leveraged advanced resources from WatSeq project, such as genes having Watkins‐unique variation. Watkins‐unique variant genes are those that have allelic variation in the Watkins landraces but not in cultivars (Cheng *et al*., 2024). Genes in the prioritized QTL were extracted from wheat IWGSC_RefSeq_v2.1 using the BioMart function of EnsemblPlants. Gene expression data for spike and grain at developmental stages important for N uptake and GPC accumulation were acquired from the Wheat Expression Database based on published data (Choulet *et al*., 2014). GWAS for GW, GL and grain area (GA) was performed following the same method as GPC and regions consistent across all three environments were prioritized. Haplotypes in these regions were tested for their effect on GW, GL and GA as well as GPC.

### Independent QTL validation in a biparental cross between Paragon × W0075

To independently validate our GWAS results, we analysed a single‐seed‐descent population (F_5_) of RILs derived from a cross between Paragon × W0075. W0075 is a spring wheat Watkins landrace genotype of AG 3 originating from Iran (Cheng *et al*., 2024). W0075 carried the beneficial haplotype at *SGPH‐A1* for reduced single‐grain protein heterogeneity (SGPH) and segregated from Paragon at GW QTL on chromosome 6A. On this 6A locus, the major haplotype significantly increased GW and moderately increased GPC in W0075 compared to the minor haplotype in Paragon, making this population suitable for independently validating our results. A field experiment was conducted in three replicates using a completely randomized design at the University of Nottingham farm, Sutton Bonington Campus, Leicestershire, UK (52°50′07.4″N 1°15′11.3″W) in 2024/2025. The soil type was a medium sandy loam. The sowing date was 15 October 2024 and the seed rate 300 seeds m^−2^. The soil mineral N in mid‐March was 72 kg N ha^−1^ and 180 kg N fertilizer was applied as ammonium nitrate prill. Herbicides, fungicides and pesticides were applied as necessary. The plot size was 6 × 1.63 m. In all plots, a plot combine was used to machine‐harvest the grain yield from a plot area of at least 5 m^2^, and values adjusted to 100% dry matter. Single grain analysis was conducted on samples of machine‐harvested grain for each plot using HSI to determine GPC as described above. HSI data were used to determine grain size traits, and a previously reported power model ($\hat{y}$ = *x* ^1.32^, where $\hat{y}$ and *x* represent weight and area of a single wheat grain, respectively (Kim *et al*., 2021)) was used to determine GW. Genotyping data from the 35 K axiom array were downloaded from Grassroot Data Repository (Earlham Institute, UK; Toronto International Data Release Workshop, 2009). QTL analysis was conducted with the rqtl package in R‐4.4.1 following a semi‐automated pipeline publicly available as part of the WatSeq project (Cheng *et al*., 2024; R Core Team, 2024).

## Results

### Hyperspectral imaging enables phenotyping for grain protein heterogeneity

We developed a model that predicts GPC from HSI data by examining 248 single grains that represent 124 genotypes of the Watkins landrace collection, with crease‐up (ventral) and crease‐down (dorsal) images taken of each grain, including the germ end (496 images; Fig. 1a–e). Ground‐truth data of protein content were collected for the imaged grains using the Dumas Combustion method, and protein levels in individual grains ranged from 8 to 20% (Supporting Information Fig. S1). All four PLSR models were tested on the two independent test sets (one with crease‐up grains and second with crease‐down) to select best parameters for predicting GPC from different grain orientations. M2 performed best with coefficient of determination (*R* ^2^) of 0.84 and root mean square error (RMSE) of 0.99 on crease‐down grains, and *R* ^2^ 0.91 and RMSE 0.74 on crease‐up grains (Table S1). Overall, all models performed better on the test set with crease‐up grains compared to crease‐down, suggesting grain orientation influences HSI‐based calibrations. Potential reasons for differences in calibration owing to grain orientation could include surface chemistry of the grain allowing HSI to cover more surface area on the ventral side and exposing higher protein deposits near the aleurone layer, or subtle differences in distance to scanner when grains are placed in different orientations. For high‐throughput phenotyping where the aim is to capture broad panel‐level variation, such differences may not be critical, but at experimental scale if grain‐to‐grain difference is desirable, we suggest using crease‐up grains during scanning.

**Fig. 1:**
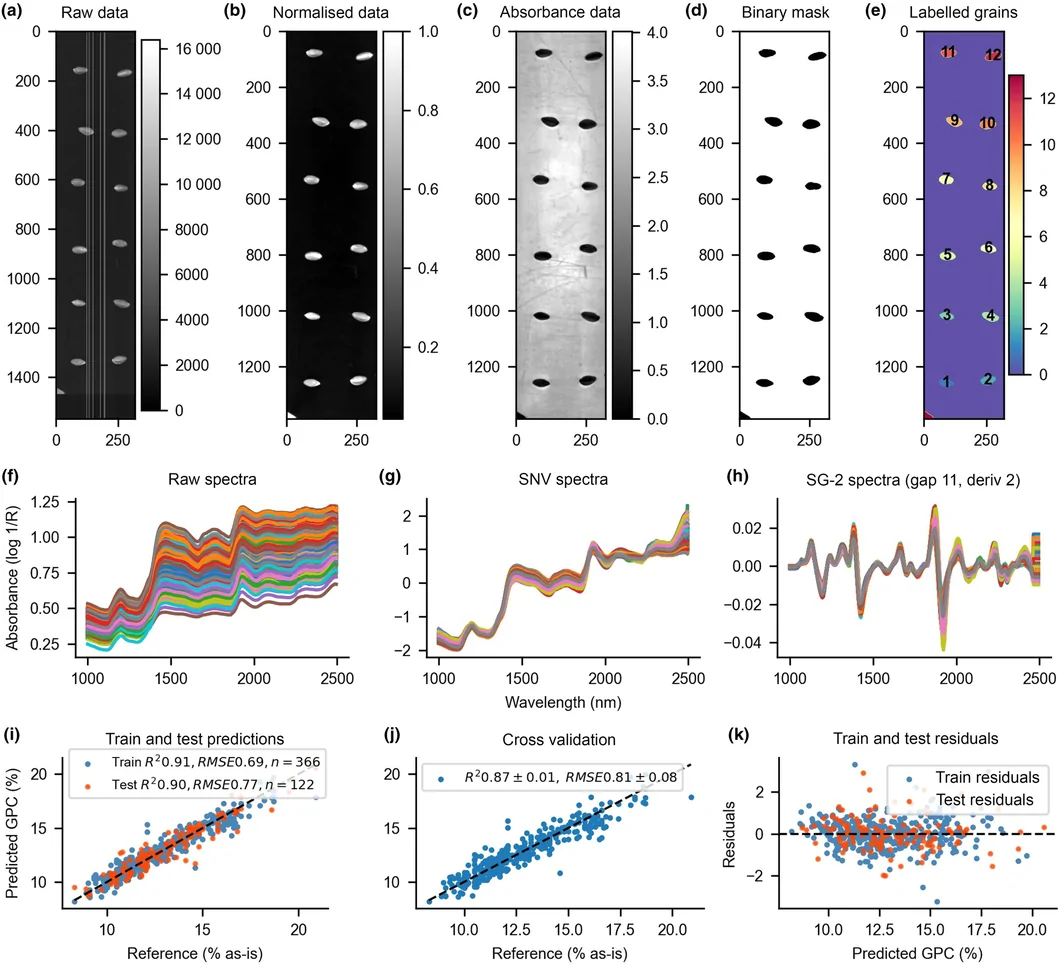
Hyperspectral imaging and machine learning‐based single‐grain protein prediction model in the Watkins wheat (*Triticum aestivum*) landraces. (a–e) Spectral data acquisition: raw data were normalized using dark and white references, converted to absorbance (log 1/reflectance) to increase the contrast, a binary mask generated using Otsu's thresholding method, and grains labelled uniquely for identification. All five panels show the same representative scan of 12 grains; the axes are image pixel coordinates, and each colour bar applies to the panel it accompanies. (a) Raw reflectance recorded by the detector, in counts. (b) The same image after normalisation against dark and white references, giving relative reflectance on a 0–1 scale. (c) Normalized data expressed as absorbance, log 1/reflectance. (d) Binary mask derived from the absorbance image, with grain pixels black and background white. (e) Connected grain objects labelled uniquely; the colour bar gives the object index. (f–h) Spectral preprocessing: original spectra were pretreated by standard normal variate (SNV) and Savitzky–Golay second derivative to obtain best calibration. Each trace is the mean spectrum of one grain across 1000–2500 nm. (f) Raw absorbance spectra, log 1/R. (g) The same spectra after SNV transformation, which centres and scales each spectrum individually and so removes multiplicative scatter. (h) The SNV spectra after a Savitzky–Golay second derivative with a gap of 11 points. (i) Partial least‐squares regression fitted on a training set of 366 grains and tested on 122 grains. Predicted grain protein content (GPC) is plotted against reference GPC determined by Dumas combustion; the dashed line is the 1 : 1 line. Nine latent variables were retained. Eleven were identified as optimal (Fig. S2), but RMSE was unchanged between nine and 11, so the smaller model was chosen to guard against overfitting. Training *R* ^2^ = 0.91, RMSE = 0.69; test *R* ^2^ = 0.90, RMSE = 0.77. (j) Calibration validated using fivefold cross‐validation, showing *R* ^2^ and RMSE values similar to the test set. Values are the mean ± SD across the five folds (*R* ^2^ = 0.87 ± 0.01, RMSE = 0.81 ± 0.08). The dashed line is the 1 : 1 line. (k) Training and test set residuals show random distribution along zero without any patterns indicating non‐biased calibration. Residuals are predicted minus reference GPC, in percentage points, plotted against predicted GPC.

After selection of the optimal modelling parameters, all data were recombined for the development of final model. The optimum number of latent variables for the PLSR model were selected based on a cross‐validation approach, which resulted in 11 components as optimum for model development. However, the RMSE was essentially unchanged between 9 and 11 and increased steadily after 13 (Fig. S2); hence, 9 components were selected to limit overfitting, a known risk for PLSR models that are subsequently applied to samples that did not contribute to model training (Gowen *et al*., 2011). The raw spectra were normalized by standard normal variate (SNV) followed by Savitzky–Golay (SG) 2nd derivative before fitting the calibration (Fig. 1f–h). The final model trained with 366 grains and tested on 122 grains yielded *R* ^2^ 0.91 and RMSE 0.69 on training set, *R* ^2^ 0.90 and RMSE 0.77 on test set and *R* ^2^ 0.87 ± 0.01 and RMSE 0.81 ± 0.08 for cross‐validation (Fig. 1i,j). Residuals for the training and test data sets distributed randomly along the zero line without showing any trend, and only a few outliers, validating the model's performance (Fig. 1k). Taken together, this model accurately measures protein content from single wheat grains and performs consistently on different subsets of unseen data with prediction capabilities that exceed those achieved by previous HSI‐based models (Caporaso *et al*., 2018; Xuan *et al*., 2024).

### The Watkins collection displays significant grain protein variation

Single‐GPC was predicted across two large field trials: *c*. 8000 grains representing 685 genotypes grown in 2020 at John Innes Centre (JIC; Norwich, UK) and *c*. 7000 grains from 596 genotypes grown in 2022 at Rothamsted Research (RRes; Harpenden, UK) were analysed (Table S2). Considerable variation was observed both among genotypes for mean GPC and the heterogeneity of protein content, which is defined here as within‐genotype CV% between single grains (Fig. 2a,b). Notably, genotypes with higher mean GPC tended to display lower within‐genotype heterogeneity. The inverse relationship between GPC and within‐genotype CV% was weak but significant (*r =* −0.27, *P* < 0.001 in both environments; Fig. 2c). Critically, a relationship of this magnitude may not imply a shared genetic signature. As heterogeneity is expressed as CV% (SD/mean), we reconstructed each genotype's underlying within‐grain SD and regressed it on mean GPC. The absolute SD did not decrease as mean GPC rose – it was statistically independent of marginally increasing (*r = c*. 0.2, *P* < 0.001 in both environments; Fig. S3). High GPC genotypes are therefore not intrinsically more uniform in absolute terms in our study. We also identified genotypes with contrasting GPC despite similar levels of heterogeneity, as well as ones with varying heterogeneity but comparable GPC, indicating these traits are genetically distinct (Fig. 2d). The CV% data showed a slight positive skew and departed from normality (Shapiro–Wilk W = *c*. 0.98, *P* < 0.001, skewness < 0.5 in both environments), although the significance of the test reflects the large sample size rather than a substantial departure, and excess kurtosis was negligible in both environments (0.03 and 0.28), indicating no heavy tails (Fig. S4). Analysis by AG revealed geographical and historical patterns of variation (Fig. 2e); AG1 exhibited high GPC coupled with low heterogeneity, while AG2 was distinguished by lower GPC and higher heterogeneity. These results have direct implications for the selection of superior lines in breeding programmes, with our data indicating the Watkins collection harbours variation absent from modern breeding that can enhance grain quality traits.

**Fig. 2:**
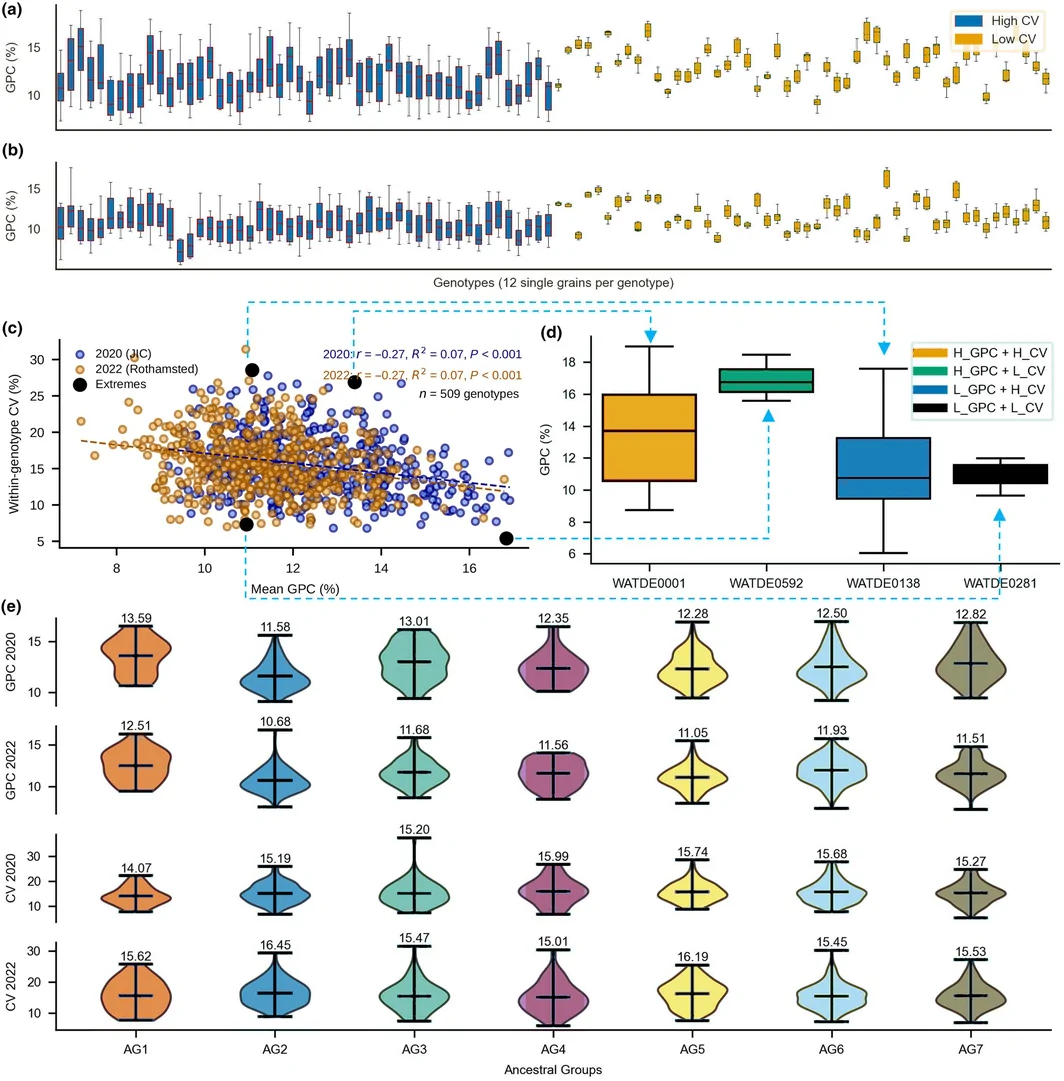
Watkins wheat (*Triticum aestivum*) landraces show highly variable grain protein content (GPC) and its heterogeneity. Protein content predicted from *c*. 8000 single grains in 2020 (a) and *c*. 7000 single grains in 2022 (b). Each box summarizes the 12 single grains of one genotype: the box spans the interquartile range; the centre line is the median and the whiskers extend to the most extreme values; outliers are not plotted. Genotypes are coloured by within‐genotype heterogeneity: red, high coefficient of variation (CV); green, low CV. Only 50 genotypes from each experiment are shown due to limited space; full data are included in the Supporting Information Table S2. (c) Mean GPC of each genotype plotted against its within‐genotype heterogeneity, expressed as the coefficient of variation (CV, %), for 2020 (JIC, blue) and 2022 (Rothamsted, red); *n* = 509 genotypes. GPC ranges between 5 and 20%, while average distribution across genotypes ranges between 8 and 17%. GPC and CV% show a weak but significant inverse correlation. Dashed lines are least‐squares fits within each year (2020: *r* = −0.27, *R* ^2^ = 0.07, *P* < 0.001; 2022: *r* = −0.27, *R* ^2^ = 0.07, *P* < 0.001). The large green points mark the four extreme genotypes shown in (d), and the blue dashed arrows link each of them to its box in that panel. (d) Examples of genotypes with high GPC : high heterogeneity (red), high GPC : low heterogeneity (green), low GPC : high heterogeneity (blue) and low GPC : low heterogeneity (black) on single grain basis. The four accessions are, in the order plotted, WATDE0001, WATDE0592, WATDE0138 and WATDE0281, each summarizing 12 single grains; box, interquartile range; centre line, median; whiskers, most extreme values. (e) The four rows show (from top to bottom) the distribution of GPC in 2020 and 2022 and CV in 2020 and 2022, respectively, in different ancestral groups. Ancestral groups AG1–AG7 are those resolved by population structure at *K* = 7 (Cheng *et al*., 2024). Each violin is a kernel density of the genotype values in that group; the vertical bar spans the full range and is capped at the minimum and maximum, and the horizontal crossbar marks the group mean, whose value is printed above each violin. AG1 has high GPC and low heterogeneity, whereas AG2 has lower GPC and higher heterogeneity.

### Grain size and weight are weakly correlated with protein content in landraces

Analysis of the Watkins landraces revealed that GPC exhibited a negative, though weak, correlation with GW, GL and GA in this collection (*P* < 0.05, *r* ≤ –0.2; Fig. 3). Because ancestral subgroups differ systematically in both traits, we partitioned this relationship into between‐ and within‐group components using the admixture proportions. AG accounted for 13.1% of variance in GPC and 19.8% in GW, and the correlation between group means was strong (*r* = −0.75). This between‐group variance was largely attributable to AG1, which combined the highest GPC with the lowest GW (excluding AG1, between‐group *r* = −0.36, *P* = 0.49). Within groups, the association was weakened but remained detectable (*r* = −0.14, *P* = 0.002; equivalent after continuous adjustment for admixture) and the same pattern held when each environment analysed separately (Fig. S5). Therefore, the weak negative association between GPC and GW in this collection is driven partly by divergence among AGs rather than by a uniform coupling of the two traits. However, the relationship shown here is for GW and does not indicate plot‐scale yield.

**Fig. 3:**
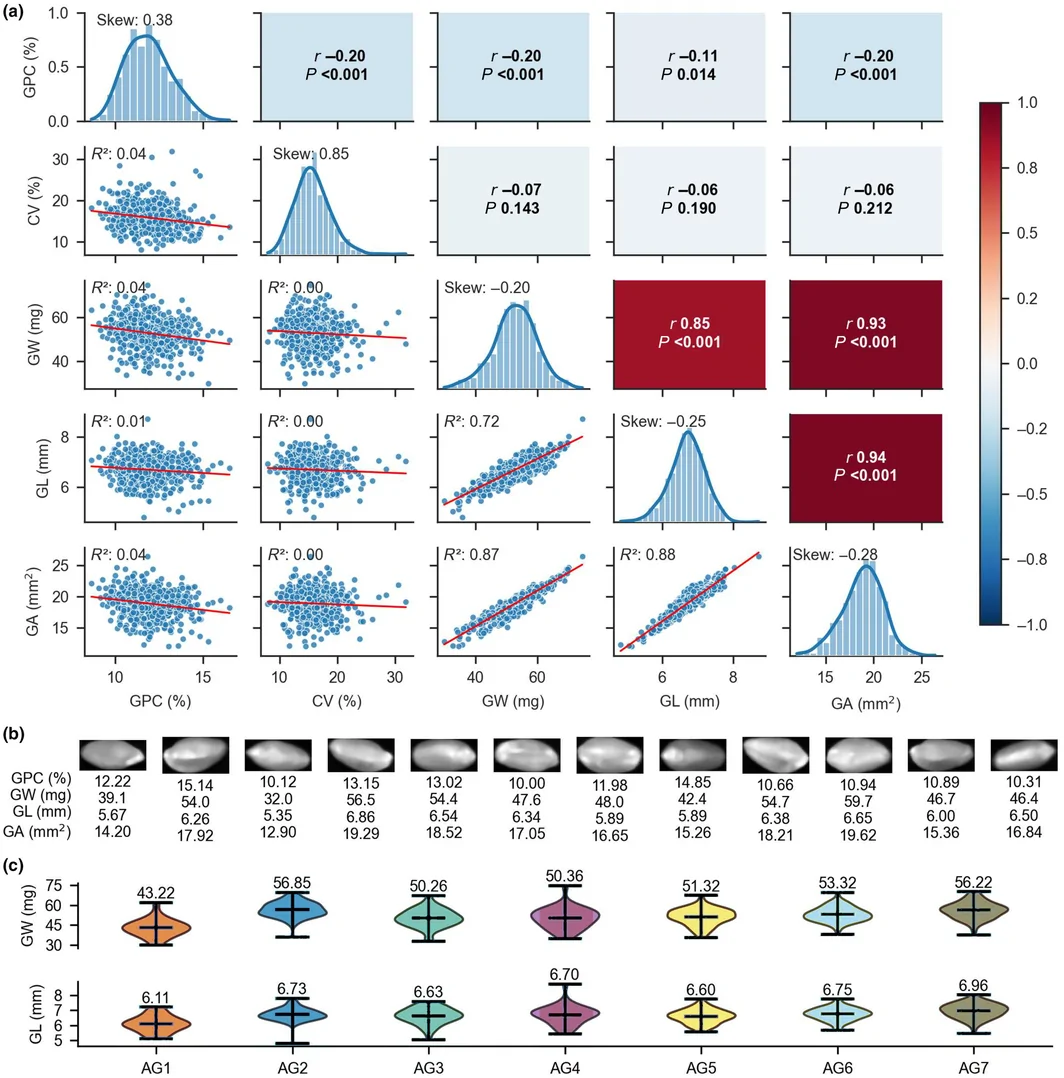
Correlations between grain protein content (GPC) and its heterogeneity, and morphological traits in the Watkins wheat (*Triticum aestivum*) panel. (a) Lower triangle shows linear regression between traits. *R* ^2^ denotes coefficient of determination for the linear relationships. The five traits are grain protein content (GPC, %), within‐genotype GPC heterogeneity (CV, %), grain weight (GW, mg), grain length (GL, mm) and grain area (GA, mm^2^); each point is one genotype (*n* = 509) and the red line is the least‐squares fit. Middle panel cutting the lower and upper triangles shows histograms of traits along with skewness of distribution. Upper triangle shows Pearson's correlations between traits. The heat signature shows strength of correlations (colour bar) and *P* values show significance level. The colour bar runs from *r* = −1 to *r* = +1. (b) Single grain images captured by the hyperspectral camera, displayed at a static band as example. The 12 grains are all from one genotype and are shown solely to illustrate a grain scan alongside the phenotypes printed beneath each image; the particular wavelength band and the identity of the genotype are not material to the point being made. Values for GPC, GW, GL and GA demonstrate that GPC in the Watkins panel is not correlated with these traits, such that grains with lower GPC may have greater length or area or vice versa. (c) Distribution of GW and GL in different ancestral groups shows AG1 to have higher GPC (Fig. 2), lower GW and GL. Ancestral groups AG1–AG7 are those resolved by population structure at *K* = 7 (Cheng *et al*., 2024). Violins are kernel densities of the genotype values; the vertical bar spans the full range and is capped at the minimum and maximum, and the horizontal crossbar marks the mean, whose value is printed above each violin.

Furthermore, GW showed a strong positive linear relationship with both GL and GA, indicating these morphological features were closely linked (Fig. 3a). We harnessed the HSI‐derived spectral and morphological data to construct a predictive model for GW, achieving a high coefficient of determination (*R* ^2^ 0.88) and a low RMSE (3.54 mg GW) that underscores the robustness and accuracy of HSI‐based phenotyping in simultaneously predicting GPC and GW (Fig. S6). Our integration of spectral and morphological features markedly improved the calibration compared with previous attempts that relied solely on HSI spectra to predict GW, for example *R* ^2^ 0.62 and RMSE 7.44 (Caporaso *et al*., 2018). Analysis of GPC across grains with varying lengths, weights and area supports the observation that GPC was largely independent of these traits (Fig. 3b). Variation in GW and GL was also evident among AGs within the Watkins collection. AG1 stands apart by exhibiting higher GPC levels alongside lower GW and GL compared to other groups. AG1 exhibited high GPC coupled with low heterogeneity; however, this was accompanied by lower GW and GL (Fig. 3c), potentially reflecting the dilution effect where smaller grains concentrate protein. Later observations showed that variation in GPC and heterogeneity was more informatively stratified by geographical origin than by AG, which directed our focus towards the geographic distribution of beneficial haplotypes. These results highlight distinct genetic profiles within the collection, relevant for targeted breeding efforts.

### GWAS identifies novel GPC loci in the Watkins collection

We selected loci with –log_10_ *P* value ≥ 5 for initial screening which identified 152 chromosomal regions showing significant association with GPC (Table S3). GWAS computation methods suggest that ignoring multiple testing correction can increase Type‐I error rate, resulting in the detection of false positives. On the other hand, too stringent testing corrections such as Bonferroni's method often increase Type‐II error rate, resulting in false negatives, thereby overlooking vital information, which is critical for a trait like GPC which is controlled by many small effect environment‐dependent alleles. The probability of an association to truly exist is not represented only by the *P* value, but also the sample size and minor allele frequency in the population (Kraft *et al*., 2009). A significant association in one population could appear non‐significant in another at the same threshold. Therefore, the initial threshold was used based on Cheng *et al*. (2024) for the same diversity panel and complemented with further analysis to increase the confidence in candidate loci.

Of these 152 loci, 22 identified in at least two independent environments were selected for deeper investigation (Table S4). To refine the QTL for further investigation, we cross‐referenced them with QTL identified by Cheng *et al*. (2024) to be associated with other grain N‐related traits, which integrated bi‐parental linkage, a natural GWAS panel (827 Watkins +224 cultivars) and a NAM‐GWAS panel derived from Watkins × Paragon crosses. This comparison identified 585 corresponding QTL in linkage populations, 70 in the natural GWAS panel and 194 in NAM‐GWAS panel (Tables S5–S7). Through these overlapping QTL, the candidate list was refined to six loci (1D, 2B, 5D, 6D, 7A and 7D) based on robust association signals and supportive evidence, such as highly significant –log10*p* values (e.g. 1D: 7.23; 6D: 7.82), number of significant SNPs (e.g. 122 SNPs on 6D) and cross‐validation with GPC‐related QTL from previous studies (Fig. 4a; Table S8). Contribution to the phenotypic variance explained (PVE) for each locus was determined using multiple linear regression, sequentially incorporating panel structure and SNPs as predictors (Fig. 4b). Among these, the minor haplotype at 6D was noteworthy for improving GPC without a negative effect on GW (Fig. 4c–g), whereas the minor haplotype at 7D increased GPC but reduced GW (Fig. 4h–l). Genotypes carrying these advantageous minor haplotypes were primarily distributed in East Asian and former Soviet regions, with additional beneficial haplotypes identified in Spanish lines – findings that echo the historical movement of germplasm via the Maritime routes connecting Asia, Africa and Europe over the late 19th–early 20th centuries (see Discussion).

**Fig. 4:**
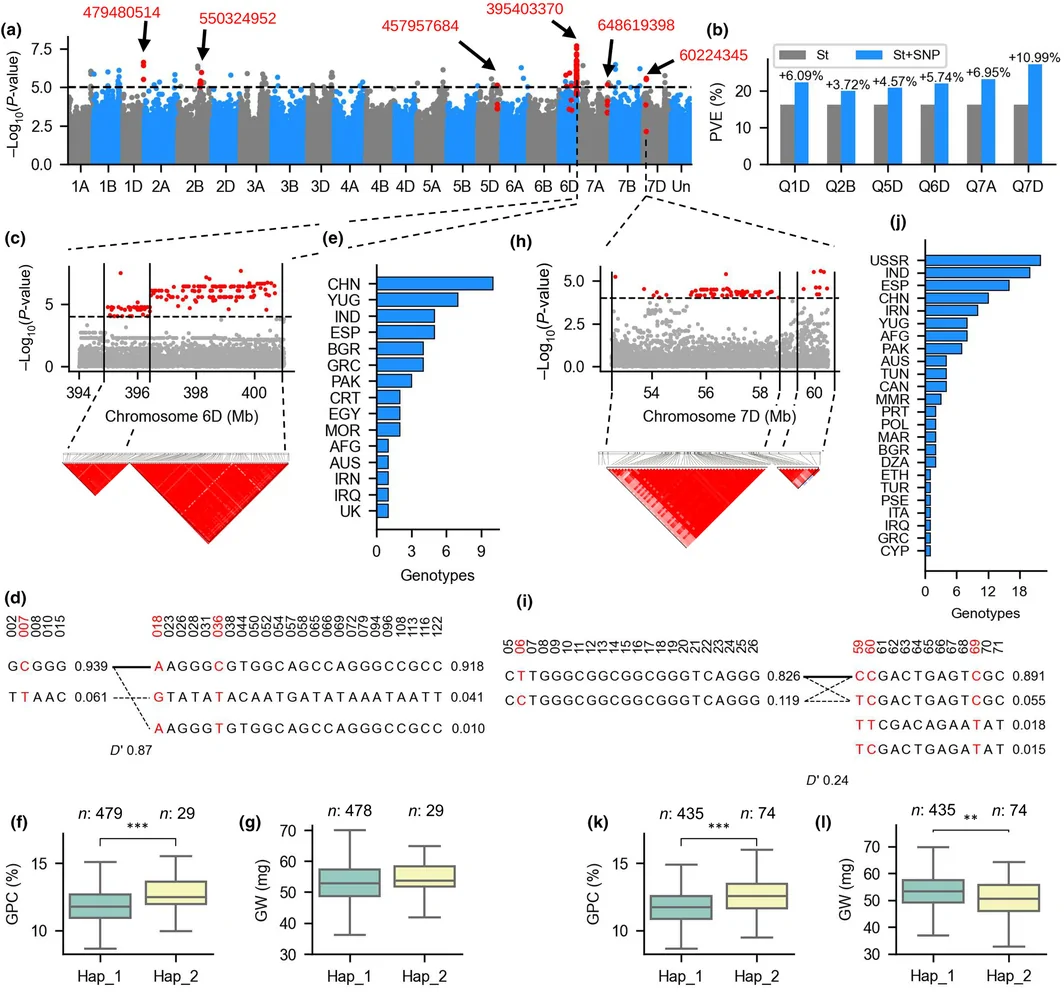
GWAS identifies new loci associated with grain protein content (GPC) in Watkins wheat (*Triticum aestivum*) landraces. (a) Manhattan plot generated from a linear mixed model (q + k); the dashed line indicates –log_10_ *P* > 5 significance, with red single nucleotide polymorphisms (SNPs) significant across multiple environments. Six major loci linked to GPC are marked by black arrows. Alternating grey and blue shading distinguishes consecutive chromosomes, and ‘Un’ holds markers that could not be anchored to a chromosome. The number beside each arrow is the physical position, in base pairs, of the peak SNP at that locus. (b) Proportion of GPC heritable variance explained by the six loci, illustrating the increased phenotypic variance explained (PVE) when SNPs are incorporated into the baseline model. Grey bars (St) are the structure‐only baseline model and blue bars (St + SNP) the same model with the significant SNP added; the value above each pair is the additional PVE contributed by SNPs. Loci are labelled by trait and chromosome. (c) Detailed view of the GPC locus on 6D, showing two LD blocks. The local Manhattan plot is drawn with a –log_10_ *P* threshold of 4 (dashed line) rather than the genome‐wide threshold of 5, so that SNPs in linkage with the significant peak are retained for the linkage disequilibrium (LD) and haplotype analyses; vertical lines delimit the two blocks. The triangle beneath shows pairwise *r* ^2^ between SNPs, deeper red indicating stronger LD. (d) Haplotypes found within LD blocks at 6D and their panel frequencies, with tag SNPs highlighted in red. The numbers above each block index the SNPs it contains. Connector lines show the *D*′ relationship between haplotypes of the two blocks: solid lines indicate stronger and dashed lines weaker linkage, with the value given beneath (*D*′ = 0.87). (e) Number of genotypes carrying Hap_2 at 6D, by country of origin (three‐letter codes). (f, g) Influence of 6D haplotypes on GPC and grain weight. Boxes span the interquartile range, centre lines are medians and whiskers extend to the most extreme values; outliers are not plotted. *n* above each box is the number of genotypes carrying that haplotype. (h) Detailed view of the GPC locus on 7D displaying two LD blocks. Plotted and annotated as in (c). (i) Haplotypes present in 7D LD blocks; in block 1, only 22 out of 51 SNPs are shown because the two haplotypes differ at a single position, SNP 06, the remaining SNPs being invariant between them. Connector lines and *D*′ as in (d). (j) Distribution of genotypes containing Hap_2 at 7D, by country of origin. (k, l) Effect of 7D haplotypes on GPC and grain weight. Boxplot conventions as in (f, g). Box plot asterisks: **, *P* < 0.01; ***, *P* < 0.001 (*t*‐test). Tests are two‐sided. Haplotype frequencies do not sum to one because accessions with ambiguous calls were excluded and, in some blocks, only the most frequent haplotypes are shown.

Comparison against published QTL in elite wheat cultivars demonstrated that several of the prioritized loci are novel (Table S9). Comparison of the candidate haplotypes with the full Watkins HapMap confirmed that many beneficial haplotypes are rare or absent in modern cultivars but frequent in the Watkins landraces (Cheng *et al*., 2024) (Table S10). For instance, Hap_13 in block2552 (overlapping 2B QTL) increases N‐use efficiency by 47.70%, present in 49 Watkins landraces and only three cultivars; Hap_12 in block0299 (7D) boosts N‐utilization efficiency by 11.20%, found in 34 landraces but no cultivars; and Hap_9 in block0301 from 7D increases grain protein deviation (GPD) by 12.60%, is again absent in cultivars but detected in 45 landraces. Conversely, haplotypes leading to decreased NUE or grain nitrogen content (GNC) are dominant in elite cultivars (Table S10). Thus, the Watkins landraces represent a valuable genetic reservoir for GPC improvement.

### Watkins‐unique variant candidate genes prioritized for targeted breeding

To narrow down from loci to candidate genes, we investigated the six prioritized regions using the IWGSC RefSeq v1.1 wheat reference, identifying 602 high‐confidence protein‐coding genes (loci 1–6: 125, 190, 50, 23, 63 and 155 genes, respectively). Two complementary prioritization strategies were adopted: (a) expression analysis during key developmental stages (spike: Z32, Z39, Z65; grain: Z71, Z75, Z85) and (b) functional annotation integrated with high‐expression data. Expression profiling revealed 15 highly expressed genes per locus, including several (e.g. *TraesCS2B02G372900*, *TraesCS5D02G388700*, *TraesCS6D02G287100* and *TraesCS7D02G096900*) with strikingly high transcript abundance at Z71 and Z85 in grain (and Z32, Z39 and Z65 in spike; Fig. S7), matching periods of grain filling and GPC deposition (Shewry *et al*., 2009). Some candidate genes showed broad expression in root, stem and leaf, suggesting roles beyond GPC alone. By filtering for genes with > 1 TPM in grain, we reduced the pool to 301, of which 253 shared homologies with functionally annotated genes in rice and Arabidopsis. Focusing on genes expressed at twice the level in spike and grain vs leaf or stem yielded 84 strong candidates (Table S11). These include genes encoding a GLUCOSE‐1‐PHOSPHATE ADENYLTRANSFERASE, OLIGOSACCHARYLTRANSFERASE, LIPOOXYGENASE, GRAS (lacking DELLA), LA PROTEIN, AUXIN RESPONSIVE PROTEIN, EXPANSIN, DUF642, NAC‐ and CCAAT‐BOX transcription factors: genes linked to starch biosynthesis, lipid metabolism, cell wall modification, senescence and other processes integral to GPC accumulation. These genes represent promising candidates underlying the observed variation and provide a foundation for future functional studies.

These gene families map onto the two processes that together determine GPC: the supply of nitrogen remobilized from vegetative tissue, and the deposition of starch that dilutes it. The NAC transcription factors provide the most direct link, since the only major GPC locus cloned in wheat, *Gpc‐B1*, encodes the NAC transcription factor *NAM‐B1* (Uauy *et al*., 2006). Importantly, the candidate genes identified at the 6D and 2B loci are distinct from the known NAM homeologues underlying *Gpc‐1* and *Gpc‐2*, indicating that the loci reported here represent GPC variation not accounted for by the canonical NAM pathway. The *NAC*, *CCAAT‐box* and *glucose‐1‐phosphate adenylyltransferase* candidates idenitfied here belong to a single regulatory module recently characterized in wheat endosperm, in which an NF‐Y (CCAAT‐binding) trimeric complex represses *TaNAC019*, itself a repressor of cytosolic *ADP‐glucose pyrophosphorylase*; knockdown of this complex reduces starch while increasing gluten protein content (Chen *et al*., 2024). Because GPC is expressed as a concentration and starch constitutes *c*. 70% of wheat grain dry weight, variation acting through this module alters GPC without any change in nitrogen supply (Smidansky *et al*., 2002), a mechanism directly relevant to the weak GW–GPC relationship reported above. The expansin candidates act instead on sink capacity, as *α‐expansin* expression accompanies pericarp and endosperm expansion during early grain filling and its ectopic expression in developing grains increases GW without reduces grain number (Lizana *et al*., 2010; Calderini *et al*., 2021). The CCAAT‐box family has separately been linked to NUE in wheat (Qu *et al*., 2015), consistent with the NUE associations reported below. However, we emphasize that these candidates are discussed as preliminary intended to suggest plausible biological routes and for subsequent testing to identify causal genes.

The WatSeq project catalogued over 13 000 Watkins‐specific variant genes, offering an untapped resource for wheat improvement (Cheng *et al*., 2024). Cross‐referencing these with high‐expression candidates identified 16 (in grain) and 23 (in spike) genes with Watkins‐unique mutations. Notably, these often overlapped with major‐effect haplotype blocks: for example, block0517 on 6D (NUE up to 33%); blocks0299, 0301, 0302, 0320 and 0304 on 7D (NUE up to 44%, GNC 27%, GPD 22%; Table S12). For example, a gene on 7D was found in block0302 and associated with a 10% GNC increase, while another gene in block0340 corresponded with a 14% GPD increase. These Watkins‐unique variants, absent from elite germplasm, provide new targets for functional validation via overexpression or transgenic experiments.

### Grain protein heterogeneity has a heritable component mapping to a locus on chromosome 6A

Using a robust analytical pipeline, GWAS for GPC heterogeneity was conducted on datasets from multi‐environment trials (2020, 2022 and the average across years). To account for pronounced environmental variation typical of single grain measurements, a validation subset of 263 genotypes was also analysed. Strikingly, both approaches converged on a stable locus on the long arm of chromosome 6A (interval 532–542 Mb), with significance observed across all environments and datasets (Fig. 5a). This locus was designated *SGPH‐A1* in accordance with recent wheat nomenclature (Boden *et al*., 2023). The heritability for this trait was expectedly low compared to GPC and GW. Across the two environments, broad‐sense heritability was 0.51 for GPC and 0.80 for GW, but only 0.08 for GPC heterogeneity. The low value for heterogeneity reflects strong *G* × *E* interaction and sampling error inherent in estimating CV% from *c*. 12 grains per genotype, which accounted for the larger share of the residual variance. Correcting for this sampling component raised the estimate to *c*. 0.17, indicating that heterogeneity is predominantly under environment‐specific control. This across‐environment estimate is necessarily lower than the within‐environment SNP‐based heritability *c*. 25% in 2022 (GEMMA PVE 0.248), of which 17.7% was captured by *SGPH‐A1* (Fig. 5b); the difference between them is the *G* × *E* interaction, and it is consistent with the strong environmental sensitivity expected for a single‐grain trait. Therefore, we treat all heritability values as indicative rather than definitive, and we note that our conclusion of genetic control at *SGPH‐A1* rests on the reproducible detection of the locus rather than on the magnitude of trait heritability. Accurate heritability estimate of grain‐level heterogeneity from multi‐environment replicated trials remains the outstanding test.

**Fig. 5:**
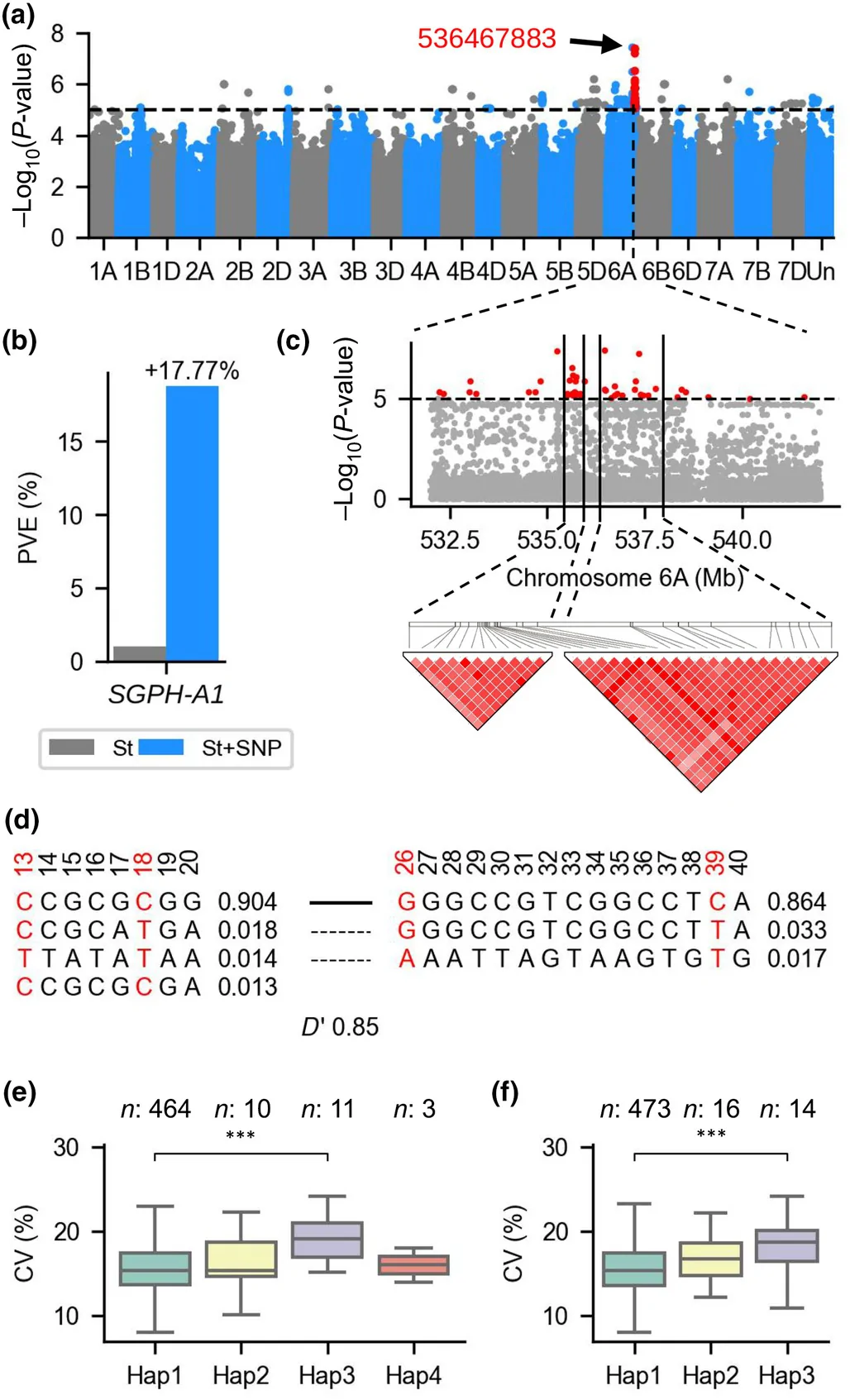
Grain protein content (GPC) heterogeneity in wheat (*Triticum aestivum*) is influenced by genetic variation. (a) Manhattan plot of GWAS for GPC heterogeneity (CV%), with significant loci indicated by SNPs in red and prioritized loci highlighted by black arrows. Association was tested with a linear mixed model. The dashed line is the genome‐wide –log_10_ *P* = 5 threshold, alternating grey and blue shading distinguishes consecutive chromosomes, and ‘Un’ holds markers that could not be anchored to a chromosome. The number beside the arrow is the physical position, in base pairs, of the peak single nucleotide polymorphism (SNP). (b) Proportion of heritable variance explained by *SGPH‐A1*. Values show additional PVE by incorporating SNPs to the baseline structure model. The grey bar (St) is the structure‐only baseline model and the blue bar (St + SNP) the same model with the peak SNP added. PVE, phenotypic variance explained. (c) Linkage disequilibrium (LD) structure of *SGPH‐A1*, defining two haplotype blocks. *SGPH‐A1* lies on chromosome 6A. The local Manhattan plot above is drawn with a –log_10_ *P* threshold of 4 (dashed line) rather than the genome‐wide threshold of 5, retaining SNPs in linkage with the significant peak for the haplotype analysis; vertical lines delimit the blocks and the triangle beneath shows pairwise *r* ^2^, deeper red indicating stronger LD. (d) Haplotype frequency distribution within each LD block. Haplotype sequences are listed with their frequency in the panel; tag SNPs are in red and the numbers above each block index its SNPs. Connector lines show the *D*′ relationship between haplotypes of the two blocks – solid, stronger; dashed, weaker (*D*′ = 0.85). (e, f) Boxplots comparing GPC heterogeneity among haplotypes in blocks 1 and 2, respectively. Boxes span the interquartile range, centre lines are medians and whiskers extend to the most extreme values; outliers are not plotted. *n* above each box is the number of genotypes carrying that haplotype. Haplotype frequencies do not sum to one because accessions with ambiguous calls were excluded and only the most frequent haplotypes are shown. Box plot asterisks: **, *P* < 0.01; ***, *P* < 0.001 (one‐way ANOVA with Tukey's HSD *post‐hoc* test).

Analysis of the 58 significant SNPs at *SGPH‐A1* revealed two distinct LD blocks of 430 kb and 1854 kb (Fig. 5c). Haplotype analysis indicated strong dominance of the major haplotype in both blocks (90.4% for block 1 and 86.4% for block 2), while minor haplotypes occurred at much lower frequency (Fig. 5d). Despite their rarity, these minor haplotypes were associated with higher GPC heterogeneity, as demonstrated by significant differences in phenotype between Hap_1 and Hap_3 in both blocks (Fig. 5e,f). Importantly, the major haplotype was consistently linked to reduced heterogeneity, suggesting that selection against rare haplotypes could be advantageous for breeding uniformity.

### Genetic determinants of grain weight and size variation in the Watkins collection

Through GWAS across three datasets (2020, 2022 and the across‐years average), we consistently detected six loci associated with GW, distributed on chromosomes 1A, 4D, 5A, 6A, 7A and 7D (Fig. 6; Table S13). Notably, the 5A locus overlapped with the previously characterized major QTL for GW and size, *QTgw.nfcri‐5A* (Wang *et al*., 2009; Brinton *et al*., 2017). Loci on 1A and 6A stood out due to their highly significant associations and were prioritized for further characterization (Fig. 6a,b).

**Fig. 6:**
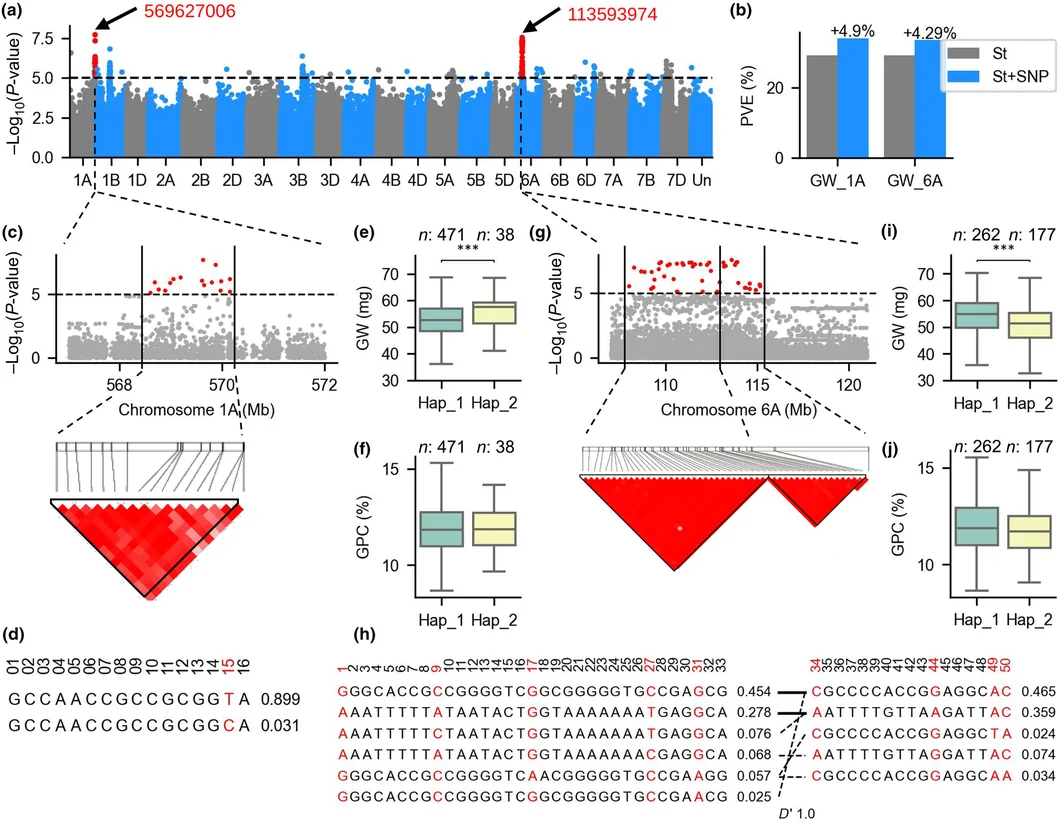
Hyperspectral imaging (HSI) with GWAS identifies novel loci associated with grain weight (GW) in the Watkins wheat (*Triticum aestivum*) landraces. (a) Manhattan plot showing linear mixed model GWAS results for GW, with significant loci indicated by single nucleotide polymorphisms (SNPs) in red and prioritized loci highlighted by black arrows. The dashed line is the genome‐wide –log_10_ *P* = 5 threshold, alternating grey and blue shading distinguishes consecutive chromosomes, and ‘Un’ holds markers that could not be anchored to a chromosome. The numbers beside the arrows are the physical positions, in base pairs, of the peak SNPs. (b) Percentage of phenotypic variance explained by the two prioritized loci, after accounting for baseline structure. Grey bars (St) show the structure‐only model and blue bars (St + SNP) the same model with the peak SNP added; the value above each pair is the additional PVE contributed by that SNP. (c) Linkage disequilibrium (LD) structure at the 1A GW locus. The local Manhattan plot above uses a –log_10_ *P* threshold of 4 (dashed line) rather than the genome‐wide threshold of 5, so that SNPs in linkage with the significant peak are retained for the haplotype analysis; vertical lines delimit the LD block and the triangle beneath shows pairwise *r* ^2^, deeper red indicating stronger LD. (d) Haplotype frequency distribution at the 1A GW locus. Sequences are listed with their frequency in the panel; tag SNPs are in red and the numbers above index the SNPs within the block. (e) Boxplot illustrating increased GW for the minor haplotype at 1A (***, *P* < 0.001, *t‐*test). Boxes span the interquartile range, centre lines are medians and whiskers extend to the most extreme values; outliers are not plotted. *n* above each box is the number of genotypes carrying that haplotype. (f) Boxplot showing no significant grain protein content (GPC) differences between haplotypes at 1A. (g) LD structure at the 6A GW locus, with two principal blocks. Plotted and annotated as in (C). (h) Haplotype frequency distribution for both LD blocks at 6A. The blocks are those delimited in (g). Sequences are listed with their frequency in the panel and tag SNPs are in red; connector lines show the *D*′ relationship between haplotypes of the two blocks – solid, stronger; dashed, weaker (*D*′ = 1.0). (i) Boxplot showing increased GW for Hap_1 at 6A Block 2 (***, *P* < 0.001, *t‐*test). Boxplot conventions as in (e). (j) Boxplot showing moderate but non‐significant increase in GPC for Hap_1 at 6A Block 2. Tests in (e), (f), (i) and (j) are two‐sided. Haplotype frequencies do not sum to one because accessions with ambiguous calls were excluded and only the most frequent haplotypes are shown.

On chromosome 1A, all significant SNPs clustered within a 1547 kb region forming a single LD block (Fig. 6c). Haplotype inference revealed a predominant major haplotype (89.9% frequency) and a rare minor haplotype (3.1%; Fig. 6d). The minor haplotype was associated with a statistically significant increase in GW (Fig. 6e) without a corresponding penalty in GPC (Fig. 6f), suggesting the absence of a trade‐off at this locus and highlighting its utility for breeding programmes targeting yield improvement. On chromosome 6A, the GW‐associated region encompassed two LD blocks spanning 4967 and 3011 kb, respectively, with a D′ value of 1.0 indicating strong linkage (Fig. 6g). Block 1 resolved into six haplotypes (frequencies: 45.4, 27.8, 7.6, 6.8, 5.7, 2.5%), while Block 2 separated into five, with the first two (46.5 and 35.9%) representing most Watkins accessions (Fig. 6h). Phenotypic analysis revealed that Hap_1 in Block 2 conferred a significant GW increase (Fig. 6i); importantly, it also showed a moderate but non‐significant rise in GPC (Fig. 6j), indicating its yield‐promoting effect does not compromise protein content, or vice versa. These results underscore that both 1A and 6A loci harbour genetic variation capable of enhancing GW without compromising GPC, offering promising avenues for marker‐assisted selection and breeding.

The exploration of grain size parameters extended beyond GW to encompass GL and GA, traits closely linked to yield potential and milling quality. Across datasets, seven loci exhibited significant associations with GL and GA (Table S14). Notably, the locus on chromosome 7D, spanning 39–44 Mb, emerged as a key pleiotropic regulator, consistently detected for both GL and GA as well as for GPC and GW. Haplotype analysis within this interval revealed Hap_2, which was linked to increased GPC and decreased GW (Fig. 4k,l), concomitantly reduced GL and GA, indicating an apparent trade‐off between protein content and grain size and weight within this region (Fig. S8). This highlights the complexity of simultaneous trait improvement and the importance of integrated breeding strategies.

### Haplotype effects at prioritized QTL are largely stable across environments

To assess whether the loci identified here would be reliable targets for marker‐assisted selection, we tested their stability across the two trial environments. For each locus, the effect of every minor haplotype relative to the major haplotype was estimated separately in each environment, and haplotype × environment interaction was tested on the within‐accession difference between environments. Across all GPC haplotype contrasts, effects estimated in the two environments were positively correlated (*r* = 0.71, *P* = 0.009) and agreed in direction in 11 of 12 cases, indicating broadly consistent behaviour. Of the five QTL prioritized here (GPC 6D and 7D; GW 1A and 6A; *SGPH‐A1*), three showed effects that were significant in both environments with no evidence of interaction. At GPC‐7D, Hap 2 (14.5% of the panel) increased GPC by 0.80 ± 0.18% in 2020 and 0.56 ± 0.22% in 2022 (*G* × *E P* = 0.91). At GW‐1A, Hap 2 increased GW by 3.15 ± 1.22 mg and 4.69 ± 1.31 mg respectively (*G* × *E P* = 0.14), while GW‐6A Hap 2 (35.5%) reduced GW by 4.42 ± 0.67 mg and 3.06 ± 0.78 mg respectively (*G* × *E P* = 0.13; Tables 1, S15).

**Table 1:** Effects of minor haplotypes at the five QTL prioritized in this study, estimated separately in each environment, and tested for haplotype × environment interaction.

Locus	Block	Haplotype	*n*	Effect 2020 (JIC)	Effect 2022 (RRes)	*P* (*G* × *E*)
GPC‐6D	Block 1	Hap 2	29	+1.47 ± 0.32***	+0.31 ± 0.26	0.004
	Block 2	Hap 2	25	+1.46 ± 0.37***	+0.36 ± 0.28	0.015
GPC‐7D	Block 1	Hap 2	74	+0.80 ± 0.18***	+0.56 ± 0.22*	0.910
	Block 2	Hap 2	19	+0.52 ± 0.38	+0.57 ± 0.27*	1.000
SGPH‐A1	Block 1	Hap 3	11	+4.35 ± 1.23**	+3.28 ± 1.33*	0.167
	Block 2	Hap 3	14	+4.73 ± 1.45*	+2.79 ± 1.64	0.558
GW‐1A	‐	Hap 2	38	+3.15 ± 1.22*	+4.69 ± 1.31***	0.142
GW‐6A	Block 2	Hap 2	177	−4.42 ± 0.67***	−3.06 ± 0.78***	0.127

Effects are the difference between the mean of the stated haplotype and that of the major (reference) haplotype at the same locus, ± SE, estimated separately in each environment. Asterisks denote the significance of each effect within its environment (*, *P* < 0.05; **, *P* < 0.01; ***, *P* < 0.001; Welch *t*‐test). *P* (*G* × *E*) tests whether the haplotype effect differs between environments, obtained by regressing the within‐accession difference between environments on haplotype, and Holm‐adjusted across all locus‐blocks tested for that trait. Only accessions phenotyped in both environments were included. The two environments differ in both site and season, so interaction cannot be attributed to site or year separately. For SGPH‐A1, the significant effect was observed for Hap 3 in both blocks compared to the reference Hap 1, hence Hap 2 is not presented. For GW‐6A, the two blocks were fully correlated (*D*′ = 1.0), hence only Hap 2 from block 2 compared to the reference Hap 1 (*n* = 262) is presented. Results for all loci tested, including those not discussed in the main text, are given in Table S15.

By contrast, the effect of Hap 2 at GPC‐6D was environment‐dependent, raising GPC by 1.47 ± 0.32% in 2020 but only 0.31 ± 0.26% in 2022 (*G* × *E P* = 0.004). At *SGPH‐A1*, Hap 3 was associated with higher protein heterogeneity in both environments (+4.35 ± 1.23 and +3.28 ± 1.33 CV%), with no significant interaction (*G* × *E P* = 0.17); however, this haplotype was carried by only 9–11 accessions, so the absence of a *G* × *E* interaction could reflect limited power (Tables 1, S15). Together, these results identify GPC‐7D, GW‐1A and GW‐6A as the most promising candidates for marker‐assisted selection and indicate that the GPC‐6D effect should be validated across a wider range of environments before deployment.

### Watkins collection provides novel genetic variation to improve grain quality

To validate the QTL identified for grain traits, we tested a population (F_5_) of recombinant inbred lines (RIL) derived from a cross between Paragon × WATDE0075 (W0075). W0075 (Hap‐1) segregated from Paragon (Hap‐2) at the 6A locus associated with GW (6A:107–121 Mb). Hap‐1 significantly increased GW with a moderate, but non‐significant increase in GPC (see Fig. 6). W0075 also he beneficial haplotype at *SGPH‐A1* (6A:532–542 Mb), resulting in reduced heterogeneity for GPC in the Watkins collection. The segregation at the 6A locus made this population suitable to validate our HSI‐derived predictions and to monitor variation in grain‐level protein content.

Plenty of SNP‐level variation was found between the two parents within QTL intervals for GPC, SGPH, GL and GW (Fig. S9). Single‐grain protein analysis through HSI across three replicates of 91 samples revealed that heterogeneity in GPC remained low and consistent across blocks (Fig. S10A,B). Average GPC in the population was *c*. 14.6%, which may be partly explained by the dry spring–early summer at the field site in 2025, with average rainfall being below average in Sutton Bonington. Despite this, the genotypes maintained representative grain yields with an overall mean of 7.85 t ha^−1^ (100% DM). The heritability estimates were improved for both traits (H^2^ 0.67 for GPC and 0.21 for CV%), perhaps due to replicated plots reducing environmental noise.

We identified two QTL for GPC on chromosomes 2B and 7A (Fig. S10C,D; Table S16); the QTL on 7A corresponded with the GWAS results. By contrast, the QTL on chromosome 2B was located *c*. 100 Mb on the Chinese Spring reference genome (RefSeq v1.1), which is separate from the GWAS peak detected near 540 Mb. This discrepancy may reflect LD differences or stronger linkage of GWAS markers to causal variants. Stable GPC QTL on 2B and 7A have been reported previously (Ruan *et al*., 2021; Nigro *et al*., 2024), supporting our findings. Even though variation in GPC heterogeneity was narrower in the RIL population (CV *c*. 7–15%), a strong QTL for SGPH was identified on 3D, explaining 24% of the 21% phenotypic variance for the trait, whereas a weak signal coinciding with *SGPH‐A1* was also identified, validating the GWAS results for this locus (Fig. S10E,F; Table S16). *SGPH‐A1* represents a novel locus, suggesting that protein uniformity within cultivars is a partially selectable trait.

Analysis of variance across field replicates showed that grain yield differed significantly between them (Fig. S11A). By contrast, GPC and its heterogeneity were consistent across replicates (see Fig. S10), suggesting that uniformity in GPC may have been supported by the beneficial haplotype at *SGPH‐A1*. Lines carrying this haplotype exhibited reduced within‐genotype variation in GPC (CV 7.31–15.34%, mean CV 10.99%) compared with the Watkins collection (CV 7.99–28.45%, mean CV 15.63%). Linear regression analyses showed no relationship between grain yield and SGPH (*y* = 0.07*x* + 6.94; *R* ^2^ = 0.024), whereas the expected negative relationship was observed between grain yield and GPC (*y* = −0.424*x* + 13.98, *R* ^2^ = 0.159), indicating that each 1% increase in GPC was associated with a 0.42 t ha^−1^ reduction in yield. However, the relatively low *R* ^2^ value showed that GPC accounted for only *c*. 16% of the variation in grain yield, indicating that it was a weak predictor of yield in this field experiment (Fig. S11B).

GL and weight showed consistent mean values across blocks (Fig. S12A,B). We identified a strong QTL on chromosome 5A (LOD score 13.83 for GL and 6.90 for GW), which explained *c*. 23% of the phenotypic variance (Fig. S12C). This QTL coincides with our GWAS peaks and the major *Qtgw.nfcri‐5A* locus, first reported in Chinese germplasm (Wang *et al*., 2009) and validated in UK germplasm (Brinton *et al*., 2017), which acts post‐fertilization to increase GL via pericarp cell expansion. Another QTL for GL was identified on 7A, colocalizing with our GWAS peaks (Fig. S12D). QTL for GW were identified on chromosomes 1A, 5A and 6A, with 5A being the same locus detected for GL (Fig. S12E–G). GW‐6A was mapped between 54.3 and 58.43 cM, with the peak SNP near 453 Mb on the Chinese Spring reference genome; the flanking interval SNPs covered 73–558 Mb (Fig. S12G). It overlaps with the centromeric 6A QTL (*c*. 480–540 Mb) reported to increase TGW through grain width (Simmonds *et al*., 2014; Simmonds *et al*., 2016). However, our GWAS in the Watkins collection also identified a peak between 107 and 121 Mb, suggesting that in addition to the well‐known centromeric QTL, the Watkins collection may present additional genetic variation to improve GW. A QTL for GPC improvement without GW penalty (or vice versa) was also recently reported on 6A between 67 and 76 Mb (Nigro *et al*., 2024).

Additionally, we identified QTL for GW on 1A, 1B (for both GL and GW) and 5B, which colocalized with previously known QTL or GWAS peaks. The signals on 1B coincide with *qKl‐1BL* (698–700 Mb), which increases GL and TGW (Qin *et al*., 2024). Overall, these results validate our GWAS findings and emphasize the use of HSI in breeding pipelines to investigate the genetic architecture of single‐grain traits, as well as multiple grain traits from the same dataset, minimizing cost and labour. The identification of both known QTL (5A, centromeric 6A, 1B) and novel signals (6A:107–121 Mb, 3D SGPH) demonstrates that Watkins landraces harbour unique allelic variation complementing elite germplasm, offering new opportunities to improve grain quality in wheat breeding.

## Discussion

Our predictive models for phenotyping protein and weight of single grains in the Watkins panel not only delivered robustness with the ground‐truth N‐based measurements but also exceeded existing calibrations in grain protein phenotyping in wheat and rice (Caporaso *et al*., 2018; Xuan *et al*., 2024). Through different spectral preprocessing techniques and data splits, we found that crease‐up grains are better captured by HSI. Therefore, the training data must include scans of grains from different orientations instead of random scanning or averaged spectra.

Grain yield and protein are generally negatively correlated in elite wheat varieties, limiting the simultaneous improvement of yield and grain quality (Tabbita *et al*., 2017). This is partly because modern breeding has focused on selection for high‐yielding and disease‐resistant varieties, and in doing so, the modern wheat cultivars have lost up to 22% nucleotide diversity (Pont *et al*., 2019). This loss of genetic diversity is evidenced by the fact that the functional alleles of a major GPC locus (*Gpc‐B1*) were present in 19th century hexaploid wheat but are absent in modern bread wheat (Asplund *et al*., 2010; Hagenblad *et al*., 2012). Landraces, due to their unique genetic makeups and low yields, could provide alleles to mitigate the yield–protein trade‐off. This was reflected in our study with GPC being weakly correlated with GW and size. The resequencing of Watkins landraces had revealed that up to 60% of the genetic diversity in the landrace collection remains untapped in modern wheats; elite cultivars comprise of only two of the seven AGs (Cheng *et al*., 2024). This supports investigations into the Watkins collection for trait improvement and adaptation. The novel QTL identified for GPC in our study on chromosomes 6D and 7D emphasized the rich diversity of the Watkins landraces. In both cases, the minor haplotype significantly improved GPC.

The 7D QTL with *c*. 11% PVE offers direct opportunity for marker‐assisted selection. The minor haplotype on this locus conferred consistent effects in both tested environments, indicating that its effect persists across environments. The minor haplotype on GPC‐6D, on the other hand, was environment‐dependent, its effect was more than fourfold larger at JIC in 2020 than at Rothamsted in 2022. Part of this difference is expected rather than biological. QTL effects estimated in the environment in which a locus was detected are systematically biased upwards, because loci are reported only when their test statistic exceeds a significance threshold (Beavis, 1998; Xu, 2003). Effect estimates obtained in independent environments are therefore the more realistic guide to what a breeder would achieve in practice, and a larger series of trials would be required to separate this expected overestimation from genuine environmental sensitivity. We observed that the Watkins genotypes with beneficial GPC haplotypes were predominantly found in East and Central Asia and extended through the Mediterranean and into Europe. It is known from the history of *Gpc‐B1* – a key QTL regulating GPC in wheat (Avivi, 1978; Joppa & Cantrell, 1990; Joppa *et al*., 1997) – that germplasm from the Vavilov Institute for Plant Genetics Resources were equipped with high GPC alleles. During the late 19th to early 20^th^ century, global trade underwent significant transformation due to industrialization, colonial expansion and advances in transportation, connecting Asia and Africa with Europe more closely than ever before. Maritime routes, especially those passing through the Suez Canal, became vital corridors for the movement of goods between the Indian Ocean and the Mediterranean. Among the many goods traded were agricultural products, including wheat grains, which likely spread from regions such as Central Asia and the Soviet territories into Europe (Donley, 2021; Tang, 2022). A high representation of genotypes carrying beneficial GPC alleles from these countries provide indications of how these alleles may have spread through trade. Introducing these novel alleles from the Watkins collection into elite germplasm could help improve grain quality.

The underlying genetics of intra‐cultivar protein variability remain poorly understood. By linking HSI with genomics, we were able to identify a stable QTL on chromosome 6A, *SGPH‐A1* (Fig. 5), indicating there may be some degree of heritability to underpin breeding for this trait. Importantly, *SGPH‐A1* did not overlap with any of the GPC QTL. Moreover, the relationship between GPC and its heterogeneity showed high GPC genotypes to have lower heterogeneity in the Watkins panel, suggesting simultaneous selection for both traits is possible. While GPC and its heterogeneity appear genetically distinct – mapped to separate QTL – the physiological underpinning of this relationship (and possible linkages with grain number) warrants further investigation. Potential physiological mechanisms leading to grain protein uniformity could be differences in spike architecture affecting spikelets per spike, grains per spikelet and synchronous grain development, improved vasculature to the developing grain, more even nitrogen distribution from source tissue, or reduced demand for nitrogen from individual grains. The predominance of the favourable haplotype in the Watkins panel (frequency *c*. 86–90%) implies that breeding for reduced GPC heterogeneity is achievable with minimal loss of genetic diversity. Nevertheless, future studies should seek to refine these associations by controlling for positional effects within spikes and tillers, as the current study relied on random single‐grain sampling. Furthermore, the genetic basis of GPC heterogeneity should be validated in modern wheat cultivars to determine the transferability of these findings. These results also lay the foundation for extending similar approaches to other traits of agronomic importance, such as GW, length or area. The *SGPH‐A1* locus on chromosome 6A emerges as a key target for future functional validation and marker‐assisted breeding for enhanced grain uniformity in wheat. The minor haplotype on *SGPH‐A1* showed consistent effects in both environments; however, its representation in small sample size (*c*. 3%) in the panel indicates the test was underpowered and warrants caution. Beyond protein uniformity, grain size and weight homogeneity are of considerable practical importance, particularly for the milling industry (Safdar *et al*., 2026). Uniform grain dimensions allow more consistent equipment calibration during cleaning and milling, reduce the proportion of grains lost as screenings and improve flour extraction rates. Preliminary observations in our data suggest heritable variation for GW heterogeneity in the Watkins panel. Therefore, further studies may be justified examining whether integrating selection for both GPC and GW uniformity could yield cultivars optimized for both nutritional quality and processing efficiency.

Our findings on GW align with and extend previous work on GW QTL in wheat. The 5A locus is consistent with the major GW and size QTL *QTgw.nfcri‐5A* (Wang *et al*., 2009; Brinton *et al*., 2017), while on 6A, other studies have mapped QTL such as *Qtkw.tamu.6A.573* (Cerit *et al*., 2023) with lower resolution (8075 SNPs) compared to the present work (*c*. 10 million SNPs). The GW interval on 6A identified here (107–121 Mb) is distinct from the previously described *TaGW‐A2* region (Simmonds *et al*., 2014; Simmonds *et al*., 2016), indicating the presence of novel, potentially Watkins‐specific alleles. Moreover, functional genomics studies targeting *TaGW2* homeologues through RNA interference revealed not only GW increases but also increments in GPC and gluten protein fractions (Zhang *et al*., 2018), paralleling the moderate GPC gains observed for haplotypes in this study. Due to high genetic density, the centromeric QTL has been difficult to narrow down to causal genes, but the identification of a distinct signal in Watkins highlights the complex genetic architecture of the GW QTL on 6A. The identification of loci conferring higher GW without a protein penalty may present an opportunity to break the conventional yield–protein trade‐off in elite backgrounds. Importantly, these loci were validated through linkage mapping in a cross of WATDE0075 with elite Paragon, forming a single‐seed‐descent population.

These results set the stage for the introgression of beneficial alleles from the Watkins collection into elite germplasm, with the prospect of enhancing productivity and end‐use quality. Future research should focus on validating these loci in elite cultivars, assessing their stability across wider genetic backgrounds and environments and determining the underlying candidate genes. Ultimately, such knowledge will inform the strategic development of wheat varieties optimized for both yield and quality, facilitating the advancement of resilient cropping systems.

## Competing interests

None declared.

## Author contributions

IDF, SAB, MJF, RAB, IRS, SG and MJH supervised the project and provided resources. LBS performed hyperspectral imaging of grains with input from IDF, MW and AC; GWAS and QTL mapping with input from SG, SAB, MJH, RAB and MJF, and conducted statistical analysis under guidance from MJF. AC and EDG assisted with grain scanning and data collection. AR, DS and MJH conducted the field trial at Rothamsted Research. CP and SG conducted the field trial at the John Innes Centre. SR and MJF conducted the validation field experiment at Sutton Bonington. LBS analysed the data, visualized results and wrote the manuscript with help from SAB and MJF. MJH, IDF, SAB, MJF, SG, IRS and MK reviewed and edited the manuscript. All authors read and approved the final version.

## Disclaimer

The New Phytologist Foundation remains neutral with regard to jurisdictional claims in maps and in any institutional affiliations.

## Supporting information


**Fig. S1** Single‐grain protein as determined by Dumas Combustion.
**Fig. S2** Determination of optimal latent variables for PLSR model.
**Fig. S3** Relationship between grain protein content and its heterogeneity.
**Fig. S4** Distribution of grain protein heterogeneity in the Watkins collection.
**Fig. S5** Relationship between protein content and grain weight in the Watkins collection.
**Fig. S6** Training a PLSR model for predicting grain weight.
**Fig. S7** Gene from prioritized loci expressed in spike and grain.
**Fig. S8** Effect of 7D locus on grain length and area.
**Fig. S9** Single nucleotide polymorphism‐level variation between Paragon and landrace WATDE0075.
**Fig. S10** Quantitative trait locus mapping for grain protein content in Paragon × WATDE0075 population.
**Fig. S11** Relationship of grain yield with protein content and its heterogeneity.
**Fig. S12** Quantitative trait locus mapping for grain length and weight in Paragon × WATDE0075 population.

**Table S1** Performance of different PLSR models on grain orientation.
**Table S2** Grain protein content data from two field trials predicted by HSI.
**Table S3** Loci associated with grain protein content in the Watkins wheat collection.
**Table S4** Candidate grain protein content loci along with corresponding quantitative trait locus (QTL) by Cheng *et al*. (2024).
**Table S5** Quantitative trait locus overlapping with grain protein content (loci in biparental populations by Cheng *et al*. (2024)).
**Table S6** Quantitative trait locus overlapping with grain protein content loci in GWAS panel by Cheng *et al*. (2024).
**Table S7** Quantitative trait locus overlapping with grain protein content loci in NAM‐GWAS panel by Cheng *et al*. (2024).
**Table S8** Multi‐environment loci associated with grain protein content in the Watkins collection.
**Table S9** Quantitative trait locus reported for grain protein content in wheat from 1997 to 2021.
**Table S10** Haplotypes from the Watkins HapMap overlapping with grain protein content loci.
**Table S11** Functional annotation of highly expressed genes at prioritized grain protein content loci.
**Table S12** Watkins‐unique variant genes expressed highly at prioritized grain protein content loci.
**Table S13** Loci associated with grain weight in the Watkins collection.
**Table S14** Loci associated with grain length and area in the Watkins collection.
**Table S15** Effects of minor haplotypes at all loci and haplotype × environment interaction.
**Table S16** Quantitative trait locus identified in Paragon × WATDE0075 mapping population.
Please note: Wiley is not responsible for the content or functionality of any Supporting Information supplied by the authors. Any queries (other than missing material) should be directed to the *New Phytologist* Central Office.

## Data Availability

The SNP data used in this study are available through Watkins & Worldwide Wheat G2B (https://wwwg2b.com/dataAvailable) and Grassroot Data Repository (https://opendata.earlham.ac.uk/wheat/under_license/toronto/WatSeq_2023‐09‐15_landrace_modern_Variation_Data/), which are publicly available under the Toronto Agreement. Semi‐automated pipeline used for QTL mapping is available through GitHub (https://github.com/JIC‐CSB/WatSeqAnalysis). A pipeline for hyperspectral analysis of wheat grains is available at: https://doi.org/10.6084/m9.figshare.31857235. Phenotypic data for single‐grain protein measurements is presented in Table S2.

## References

[nph71628-bib-0001] Asplund L , Hagenblad J , Leino MW . 2010. Re‐evaluating the history of the wheat domestication gene NAM‐B1 using historical plant material. Journal of Archaeological Science 37: 2303–2307.

[nph71628-bib-0002] Avivi L . 1978. High protein content in wild tetraploid *Triticum dicoccoides* Korn. Proceedings of the 5th International Wheat Genetics Symposium. New Delhi, India: Indian Society of Genetics and Plant Breeding, 372–380.

[nph71628-bib-0003] Bailey‐Serres J , Parker JE , Ainsworth EA , Oldroyd GED , Schroeder JI . 2019. Genetic strategies for improving crop yields. Nature 575: 109–118. 10.1038/s41586-019-1679-0 PMC7024682 31695205

[nph71628-bib-0004] Barrett JC , Fry B , Maller J , Daly MJ . 2005. Haploview: analysis and visualization of LD and haplotype maps. Bioinformatics 21: 263–265. 10.1093/bioinformatics/bth457 15297300

[nph71628-bib-0005] Beavis WD . 1998. QTL analyses: power, precision, and accuracy. In: Paterson AH , ed. Molecular dissection of complex traits. Boca Raton, FL, USA: CRC press, 145–162.

[nph71628-bib-0006] Boden SA , McIntosh RA , Uauy C , Krattinger SG , Dubcovsky J , Rogers WJ , Xia XC , Badaeva ED , Bentley AR , Brown‐Guedira G *et al*. 2023. Updated guidelines for gene nomenclature in wheat. Theoretical and Applied Genetics 136: 72. 10.1007/s00122-023-04253-w PMC10036449 36952017

[nph71628-bib-0007] Brinton J , Ramirez‐Gonzalez RH , Simmonds J , Wingen L , Orford S , Griffiths S , Haberer G , Spannagl M , Walkowiak S , Pozniak C *et al*. 2020. A haplotype‐led approach to increase the precision of wheat breeding. Communications Biology 3: 712. 10.1038/s42003-020-01413-2 PMC7689427 33239669

[nph71628-bib-0008] Brinton J , Simmonds J , Minter F , Leverington‐Waite M , Snape J , Uauy C . 2017. Increased pericarp cell length underlies a major quantitative trait locus for grain weight in hexaploid wheat. New Phytologist 215: 1026–1038. 10.1111/nph.14624 28574181

[nph71628-bib-0009] Calderini DF , Castillo FM , Arenas MA , Molero G , Reynolds MP , Craze M , Bowden S , Milner MJ , Wallington EJ , Dowle A *et al*. 2021. Overcoming the trade‐off between grain weight and number in wheat by the ectopic expression of expansin in developing seeds leads to increased yield potential. New Phytologist 230: 629–640. 10.1111/nph.17048 PMC8048851 33124693

[nph71628-bib-0010] Caporaso N , Whitworth MB , Fisk ID . 2018. Protein content prediction in single wheat kernels using hyperspectral imaging. Food Chemistry 240: 32–42. 10.1016/j.foodchem.2017.07.048 PMC5625851 28946278

[nph71628-bib-0011] Cerit M , Wang Z , Dogan M , Yu S , Valenzuela‐Antelo JL , Chu C , Wang S , Xue Q , Ibrahim AMH , Rudd JC *et al*. 2023. Mapping QTL for yield and its component traits using wheat (*Triticum aestivum* L.) RIL mapping population from TAM 113 × Gallagher. Agronomy 13: 2402.

[nph71628-bib-0012] Chen J , Zhao L , Li H , Yang C , Lin X , Lin Y , Zhang H , Zhang M , Bie X , Zhao P *et al*. 2024. Nuclear factor‐Y‐polycomb repressive complex2 dynamically orchestrates starch and seed storage protein biosynthesis in wheat. Plant Cell 36: 4786–4803. 10.1093/plcell/koae256 PMC11530772 39293039

[nph71628-bib-0013] Cheng S , Feng C , Wingen LU , Cheng H , Riche AB , Jiang M , Leverington‐Waite M , Huang Z , Collier S , Orford S . 2024. Harnessing landrace diversity empowers wheat breeding. Nature 632: 823–831. 10.1038/s41586-024-07682-9 PMC11338829 38885696

[nph71628-bib-0014] Choulet F , Alberti A , Theil S , Glover N , Barbe V , Daron J , Pingault L , Sourdille P , Couloux A , Paux E *et al*. 2014. Structural and functional partitioning of bread wheat chromosome 3B. Science 345: 1249721. 10.1126/science.1249721 25035497

[nph71628-bib-0015] Delwiche S . 1998. Protein content of single kernels of wheat by near‐infrared reflectance spectroscopy. Journal of Cereal Science 27: 241–254.

[nph71628-bib-0016] Delwiche SR . 1995. Single wheat kernel analysis by near‐infrared transmittance: protein content. Cereal Chemistry 72: 11–16.

[nph71628-bib-0017] Donley A . 2021. The fall and rise of Russian wheat. World Grain. [Online]. [WWW document] URL https://www.world‐grain.com/articles/16273‐the‐fall‐and‐rise‐of‐russian‐wheat [accessed 15 November 2024].

[nph71628-bib-0018] Gowen AA , Downey G , Esquerre C , O'Donnell CP . 2011. Preventing over‐fitting in PLS calibration models of near‐infrared (NIR) spectroscopy data using regression coefficients. Journal of Chemometrics 25: 375–381.

[nph71628-bib-0019] Hagenblad J , Asplund L , Balfourier F , Ravel C , Leino MW . 2012. Strong presence of the high grain protein content allele of NAM‐B1 in Fennoscandian wheat. Theoretical and Applied Genetics 125: 1677–1686. 10.1007/s00122-012-1943-2 22850788

[nph71628-bib-0020] Hao C , Wang L , Ge H , Dong Y , Zhang X . 2011. Genetic diversity and linkage disequilibrium in Chinese bread wheat (*Triticum aestivum* L.) revealed by SSR markers. PLoS ONE 6: e17279. 10.1371/journal.pone.0017279 PMC3041829 21365016

[nph71628-bib-0021] Harris CR , Millman KJ , Van Der Walt SJ , Gommers R , Virtanen P , Cournapeau D , Wieser E , Taylor J , Berg S , Smith NJ . 2020. Array programming with NumPy. Nature 585: 357–362. 10.1038/s41586-020-2649-2 PMC7759461 32939066

[nph71628-bib-0022] Herrera CM . 2009. Multiplicity in unity: plant subindividual variation and interactions with animals. Chicago, IL, USA: University of Chicago Press.

[nph71628-bib-0023] Hoseney RC . 1994. Principles of cereal science and technology. St Paul, MN, USA: American Association of Cereal Chemists (AACC).

[nph71628-bib-0024] Hunter JD . 2007. Matplotlib: A 2D graphics environment. Computing in Science & Engineering 9: 90–95.

[nph71628-bib-0025] Joppa L , Cantrell R . 1990. Chromosomal location of genes for grain protein content of wild tetraploid wheat. Crop Science 30: 1059–1064.

[nph71628-bib-0026] Joppa L , Du C , Hart GE , Hareland GA . 1997. Mapping gene (s) for grain protein in tetraploid wheat (*Triticum turgidum* L.) using a population of recombinant inbred chromosome lines. Crop Science 37: 1586–1589.

[nph71628-bib-0027] Kim J , Savin R , Slafer GA . 2021. Weight of individual wheat grains estimated from high‐throughput digital images of grain area. European Journal of Agronomy 124: 126237.

[nph71628-bib-0028] Kraft P , Zeggini E , Ioannidis JP . 2009. Replication in genome‐wide association studies. Statistical Science 24: 561–573. 10.1214/09-STS290 PMC2865141 20454541

[nph71628-bib-0029] Lizana XC , Riegel R , Gomez LD , Herrera J , Isla A , McQueen‐Mason SJ , Calderini DF . 2010. Expansins expression is associated with grain size dynamics in wheat (*Triticum aestivum* L.). Journal of Experimental Botany 61: 1147–1157. 10.1093/jxb/erp380 PMC2826655 20080826

[nph71628-bib-0030] March‐Salas M , Fandos G , Fitze PS . 2020. Effects of intrinsic environmental predictability on intra‐individual and intra‐population variability of plant reproductive traits and eco‐evolutionary consequences. Annals of Botany 127: 413–423. 10.1093/aob/mcaa096 PMC7988524 32421780

[nph71628-bib-0031] McKinney W . 2010. Data structures for statistical computing in python. In: Proceedings of the 9th Python in Science Conference. Austin, TX, USA: SciPy, 51–56.

[nph71628-bib-0032] Müller J . 2017. Dumas or Kjeldahl for reference analysis. Hilleroed, Denmark: FOSS.

[nph71628-bib-0033] Nigro D , Blanco E , Mangini G , Laddomada B , Sgaramella N , Signorile MA , Simeone R , Blanco A . 2024. Identifying QTL for grain protein content independent from grain yield‐related traits in durum wheat. Journal of Cereal Science 117: 103894.

[nph71628-bib-0034] Ninou E , Mylonas I , Karagianni I , Michailidou S , Tsivelikas A , Sistanis I , Avdikos I , Korpetis E , Papathanasiou F . 2022. Utilization of intra‐cultivar variation for grain yield and protein content within durum wheat cultivars. Agriculture 12: 661.

[nph71628-bib-0035] Pedregosa F , Varoquaux G , Gramfort A , Michel V , Thirion B , Grisel O , Blondel M , Prettenhofer P , Weiss R , Dubourg V . 2011. Scikit‐learn: machine learning in Python. The Journal of Machine Learning Research 12: 2825–2830.

[nph71628-bib-0036] Pont C , Leroy T , Seidel M , Tondelli A , Duchemin W , Armisen D , Lang D , Bustos‐Korts D , Goué N , Balfourier F . 2019. Tracing the ancestry of modern bread wheats. Nature Genetics 51: 905–911. 10.1038/s41588-019-0393-z 31043760

[nph71628-bib-0037] Qin R , Cao M , Dong J , Chen L , Guo H , Guo Q , Cai Y , Han L , Huang Z , Xu N *et al*. 2024. Fine mapping of a major QTL, qKl‐1BL controlling kernel length in common wheat. Theoretical and Applied Genetics 137: 67. 10.1007/s00122-024-04574-4 38441674

[nph71628-bib-0038] Qu B , He X , Wang J , Zhao Y , Teng W , Shao A , Zhao X , Ma W , Wang J , Li B *et al*. 2015. A wheat CCAAT box‐binding transcription factor increases the grain yield of wheat with less fertilizer input. Plant Physiology 167: 411–423. 10.1104/pp.114.246959 PMC4326744 25489021

[nph71628-bib-0052] R Core Team . 2024. R: A Language and Environment for Statistical Computing. Vienna, Austria: R Foundation for Statistical Computing.

[nph71628-bib-0039] Ruan Y , Yu B , Knox RE , Zhang W , Singh AK , Cuthbert R , Fobert P , DePauw R , Berraies S , Sharpe A *et al*. 2021. Conditional mapping identified quantitative trait loci for grain protein concentration expressing independently of grain yield in Canadian Durum Wheat. Frontiers in Plant Science 12: 642955. 10.3389/fpls.2021.642955 PMC8024689 33841470

[nph71628-bib-0040] Safdar LB , Fisk ID , Bhosale RA , Griffiths S , Hawkesford MJ , Boden SA , Foulkes MJ . 2026. Heterogeneity in seed traits: an overlooked breeding opportunity. Trends in Plant Science 31: 379–382. 10.1016/j.tplants.2025.12.009 41539883

[nph71628-bib-0041] Safdar LB , Foulkes MJ , Kleiner FH , Searle IR , Bhosale RA , Fisk ID , Boden SA . 2023. Challenges facing sustainable protein production: opportunities for cereals. Plant Communications 4: 100716. 10.1016/j.xplc.2023.100716 PMC10721536 37710958

[nph71628-bib-0042] Sarić R , Nguyen VD , Burge T , Berkowitz O , Trtílek M , Whelan J , Lewsey MG , Čustović E . 2022. Applications of hyperspectral imaging in plant phenotyping. Trends in Plant Science 27: 301–315. 10.1016/j.tplants.2021.12.003 34998690

[nph71628-bib-0043] Seabold S , Perktold J . 2010. Statsmodels: econometric and statistical modeling with python. SciPy 7: 92–96.

[nph71628-bib-0044] Shewry P . 2019. What is gluten—Why is it special? Frontiers in Nutrition 6: 101. 10.3389/fnut.2019.00101 PMC6625226 31334243

[nph71628-bib-0045] Shewry PR . 2007. Improving the protein content and composition of cereal grain. Journal of Cereal Science 46: 239–250.

[nph71628-bib-0046] Shewry PR , Underwood C , Wan Y , Lovegrove A , Bhandari D , Toole G , Mills EC , Denyer K , Mitchell RA . 2009. Storage product synthesis and accumulation in developing grains of wheat. Journal of Cereal Science 50: 106–112.

[nph71628-bib-0047] Simmonds J , Scott P , Brinton J , Mestre TC , Bush M , Del Blanco A , Dubcovsky J , Uauy C . 2016. A splice acceptor site mutation in TaGW2‐A1 increases thousand grain weight in tetraploid and hexaploid wheat through wider and longer grains. Theoretical and Applied Genetics 129: 1099–1112. 10.1007/s00122-016-2686-2 PMC4869752 26883045

[nph71628-bib-0048] Simmonds J , Scott P , Leverington‐Waite M , Turner AS , Brinton J , Korzun V , Snape J , Uauy C . 2014. Identification and independent validation of a stable yield and thousand grain weight QTL on chromosome 6A of hexaploid wheat (*Triticum aestivum* L.). BMC Plant Biology 14: 191. 10.1186/s12870-014-0191-9 PMC4105860 25034643

[nph71628-bib-0049] Smidansky ED , Clancy M , Meyer FD , Lanning SP , Blake NK , Talbert LE , Giroux MJ . 2002. Enhanced ADP‐glucose pyrophosphorylase activity in wheat endosperm increases seed yield. Proceedings of the National Academy of Sciences, USA 99: 1724–1729. 10.1073/pnas.022635299 PMC122258 11830676

[nph71628-bib-0050] Tabbita F , Pearce S , Barneix AJ . 2017. Breeding for increased grain protein and micronutrient content in wheat: ten years of the GPC‐B1 gene. Journal of Cereal Science 73: 183–191.

[nph71628-bib-0051] Tang KA . 2022. A Venice of the desert: the successes and failures of the Suez Canal in revitalising Mediterranean trade. In: Curli B , ed. Italy and the Suez Canal, from the Mid‐nineteenth Century to the Cold War: A Mediterranean History. Cham, Switzerland: Springer International Publishing, 93–110.

[nph71628-bib-0053] Tokatlidis IS , Tsialtas JT , Xynias IN , Tamoutsidis E , Irakli M . 2004. Variation within a bread wheat cultivar for grain yield, protein content, carbon isotope discrimination and ash content. Field Crops Research 86: 33–42.

[nph71628-bib-0054] Tokatlidis IS , Tsikrikoni C , Lithourgidis AS , Tsialtas JT , Tzantarmas C . 2011. Intra‐cultivar variation in cotton: response to single‐plant yield selection at low density. The Journal of Agricultural Science 149: 197–204.

[nph71628-bib-0055] Toronto International Data Release Workshop A . 2009. Prepublication data sharing. Nature 461: 168–170. 10.1038/461168a PMC3073843 19741685

[nph71628-bib-0056] Uauy C , Distelfeld A , Fahima T , Blechl A , Dubcovsky J . 2006. A NAC gene regulating senescence improves grain protein, zinc, and iron content in wheat. Science 314: 1298–1301. 10.1126/science.1133649 PMC4737439 17124321

[nph71628-bib-0057] Virtanen P , Gommers R , Oliphant TE , Haberland M , Reddy T , Cournapeau D , Burovski E , Peterson P , Weckesser W , Bright J . 2020. SciPy 1.0: fundamental algorithms for scientific computing in Python. Nature Methods 17: 261–272. 10.1038/s41592-019-0686-2 PMC7056644 32015543

[nph71628-bib-0058] Wang RX , Hai L , Zhang XY , You GX , Yan CS , Xiao SH . 2009. QTL mapping for grain filling rate and yield‐related traits in RILs of the Chinese winter wheat population Heshangmai × Yu8679. Theoretical and Applied Genetics 118: 313–325. 10.1007/s00122-008-0901-5 18853131

[nph71628-bib-0059] Waskom ML . 2021. Seaborn: statistical data visualization. The Journal of Open Source Software 6: 3021.

[nph71628-bib-0060] Wingen LU , Orford S , Goram R , Leverington‐Waite M , Bilham L , Patsiou TS , Ambrose M , Dicks J , Griffiths S . 2014. Establishing the A. E. Watkins landrace cultivar collection as a resource for systematic gene discovery in bread wheat. Theoretical and Applied Genetics 127: 1831–1842. 10.1007/s00122-014-2344-5 PMC4110413 24985064

[nph71628-bib-0061] Xu S . 2003. Theoretical basis of the Beavis effect. Genetics 165: 2259–2268. 10.1093/genetics/165.4.2259 PMC1462909 14704201

[nph71628-bib-0062] Xuan G , Jia H , Shao Y , Shi C . 2024. Protein content prediction of rice grains based on hyperspectral imaging. Spectrochimica Acta Part A: Molecular and Biomolecular Spectroscopy 320: 124589. 10.1016/j.saa.2024.124589 38850826

[nph71628-bib-0063] Zhang Y , Li D , Zhang D , Zhao X , Cao X , Dong L , Liu J , Chen K , Zhang H , Gao C *et al*. 2018. Analysis of the functions of Ta2 homoeologs in wheat grain weight and protein content traits. The Plant Journal 94: 857–866. 10.1111/tpj.13903 29570880

