# Supplementary material for: Harnessing genetic variation for grain quality from diverse wheat landraces
Source: New Phytol. 2026 Oct 9;252(5):1924–42. doi: 10.1111/nph.71628 (PMC13738675; doi:10.1111/nph.71628)
Supplement: Supplementary file 1 [file NPH-252-1924-s001.pdf]

New Phytologist

***New Phytologist* Supporting Information**

Article title: Harnessing genetic variation for grain quality from diverse wheat landraces

Authors: Luqman B. Safdar, M. John Foulkes, Alix Cornish, Elizabeth D. Goodman, Andrew B. Riche, David Steele, Charlie Philp, Shiva Rahimitanha, Martin Whitworth, Iain R. Searle, Matt Kerton, Malcolm J. Hawkesford, Simon Griffiths, Rahul A. Bhosale, Scott A. Boden, Ian D. Fisk

Article acceptance date: 05 September 2026

The following Supporting Information is available for this article:

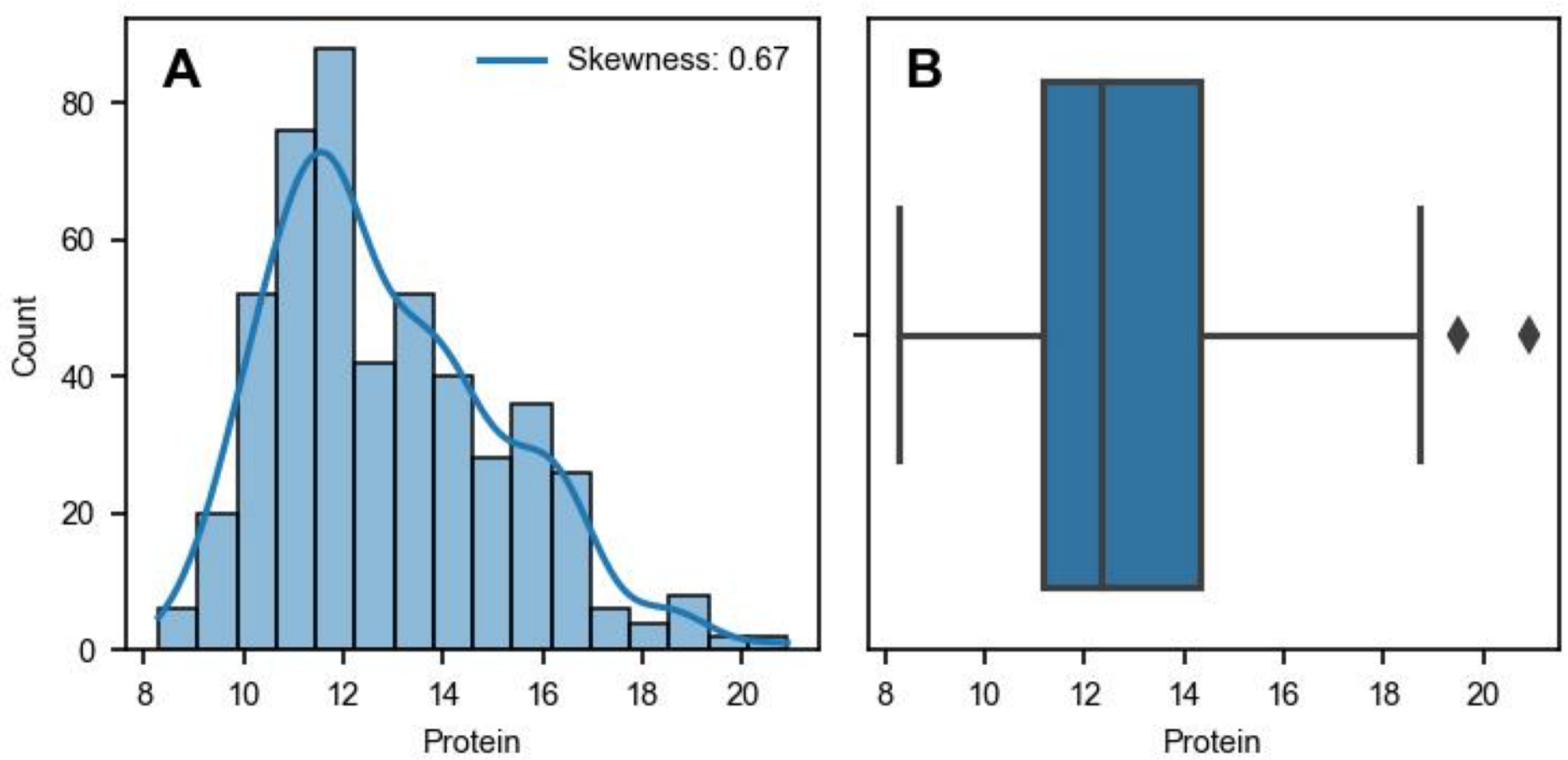

Fig. S1 Single grain protein as determined by Dumas Combustion. (A) Data distribution showing a moderately positive skewness and a tail towards the right. **(B)** Box plot of data showing a mean distribution around 12%, with two outliers. This single grain ground-truth data was collected using Dumas Combustion method and used as reference in the calibration method.

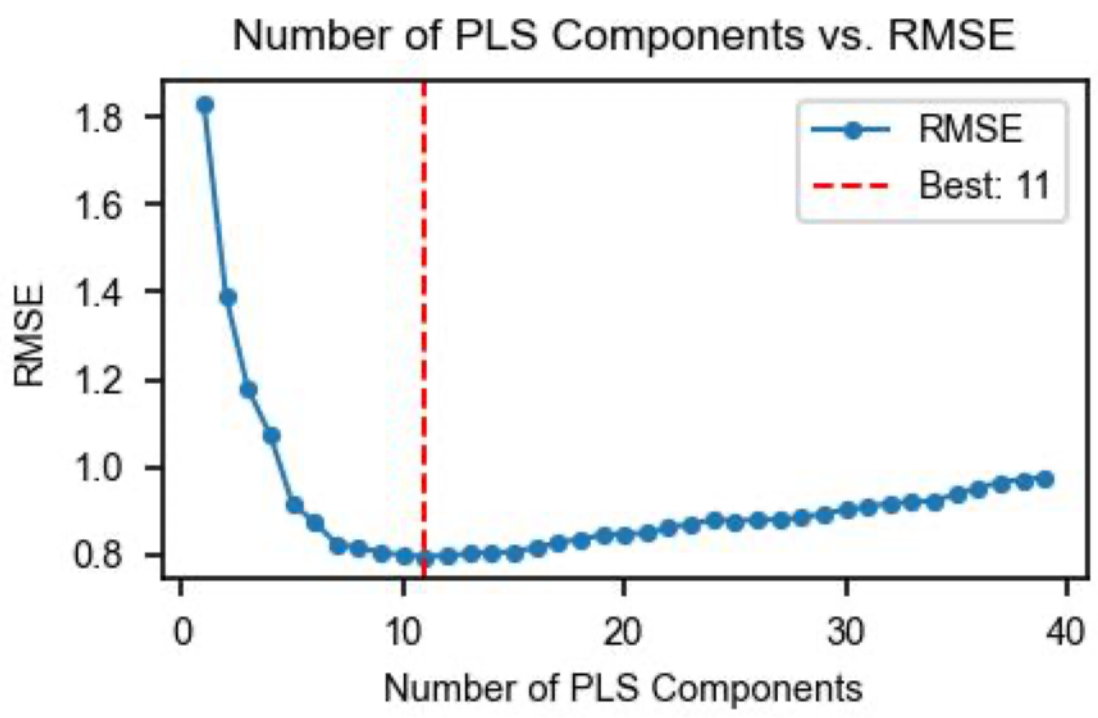

Fig. S2 Determination of optimal latent variables for PLSR model. The optimum number of components were determined by cross-validation approach. Although 11 principal components were found as optimum, the curve flattened between ~8-15 with no difference in RMSE between 9 and 11, and a steady increase after 15. Therefore, 9 components were selected for model optimization.

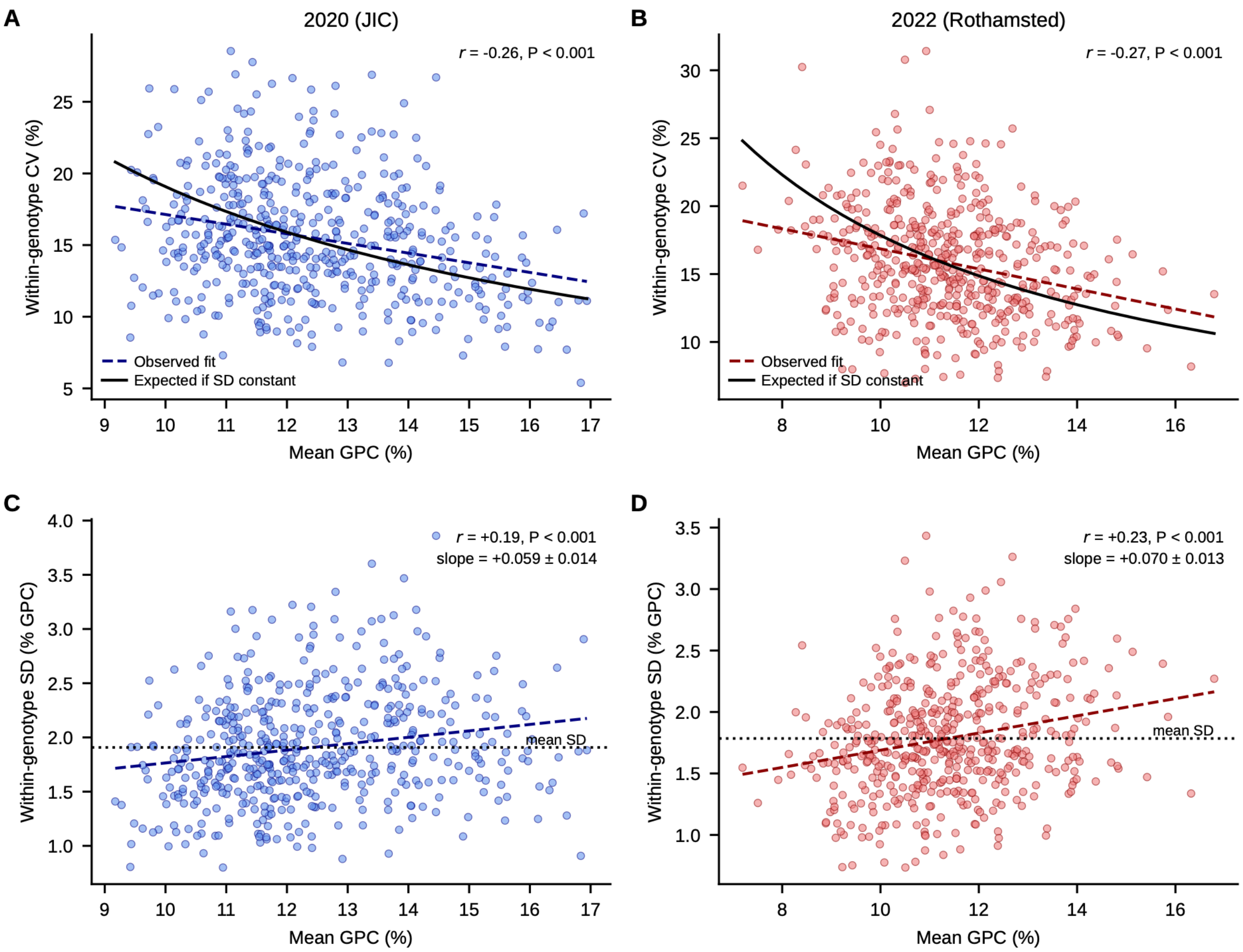

Fig. S3 The inverse relationship between mean grain protein content and within-genotype protein heterogeneity is largely a consequence of expressing dispersion as a coefficient of variation. (A, B) Within-genotype CV (%) plotted against mean GPC (%) for 2020 (JIC) and 2022 (Rothamsted). The dashed line shows the observed linear fit; the solid black curve shows the CV expected if the absolute within-genotype standard deviation were identical for all genotypes (CV = 100 × mean SD / mean GPC). The observed decline closely follows this constant-SD expectation. **(C, D)** The same genotypes plotted as absolute within-genotype standard deviation against mean GPC, with the observed linear fit (dashed) and the panel mean SD (dotted). SD does not decline as mean GPC increases, indicating that the fall in CV reflects the shared mean term in its denominator. Each point represents one genotype (n = 510 per environment); values were calculated directly from single-grain measurements. Pearson correlations: CV against mean, $r = -0.26$ (2020) and −0.27 (2022); SD against mean, $r = +0.19$ and +0.23; all $P < 0.001$.

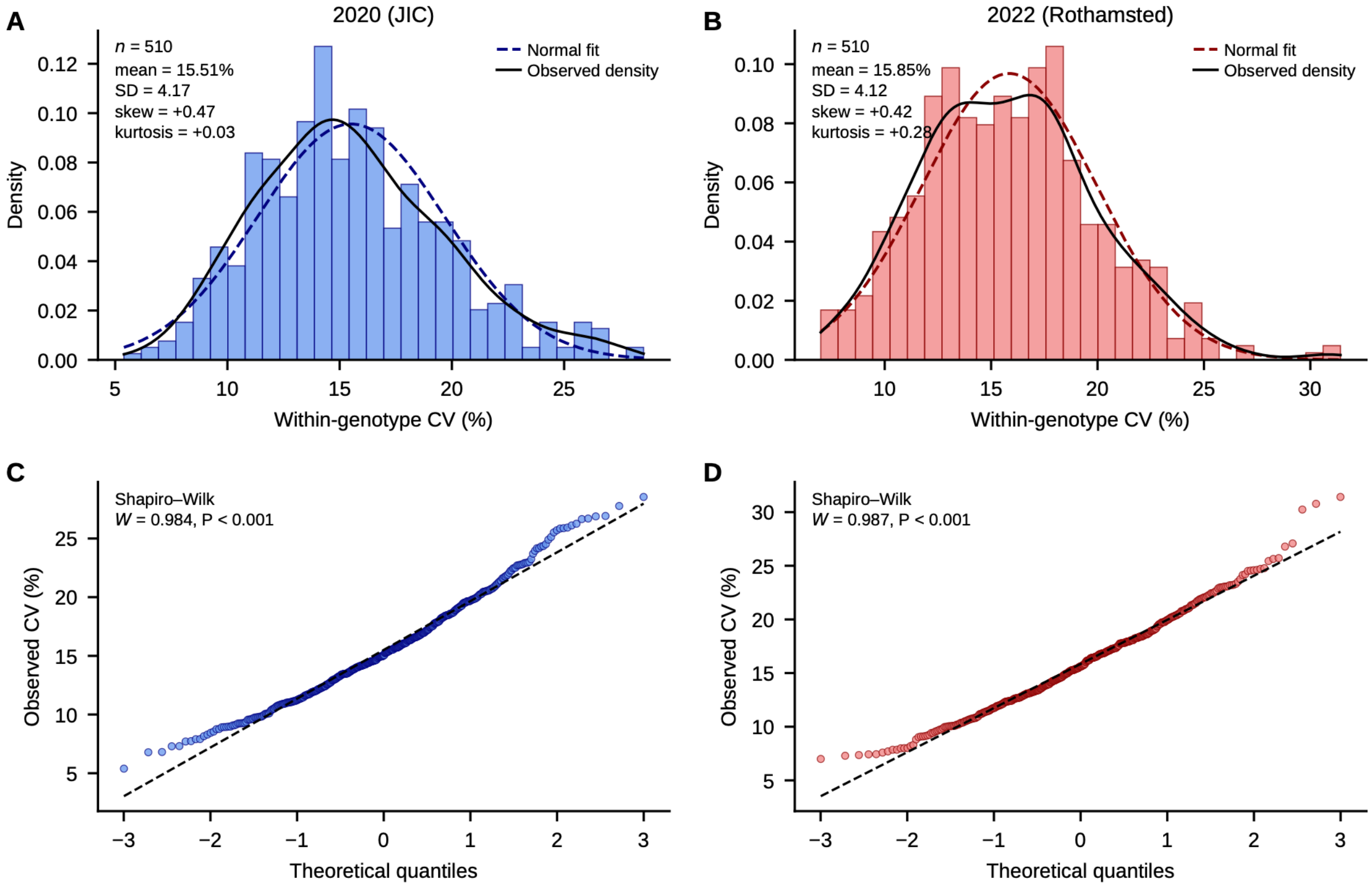

Fig. S4 Distribution of grain protein heterogeneity (CV%) in the Watkins collection. (A, B) Histograms of CV% across genotypes for 2020 (JIC) and 2022 (Rothamsted), with the observed kernel density (solid black) and a fitted normal distribution (dashed) overlaid. (C, D) Corresponding normal quantile–quantile plots; the dashed line indicates the expected relationship under normality. CV% was calculated per genotype across the single grains scored in each environment (n = 510 genotypes per environment). Summary statistics, Shapiro–Wilk test results, skewness and excess kurtosis are shown in each panel.

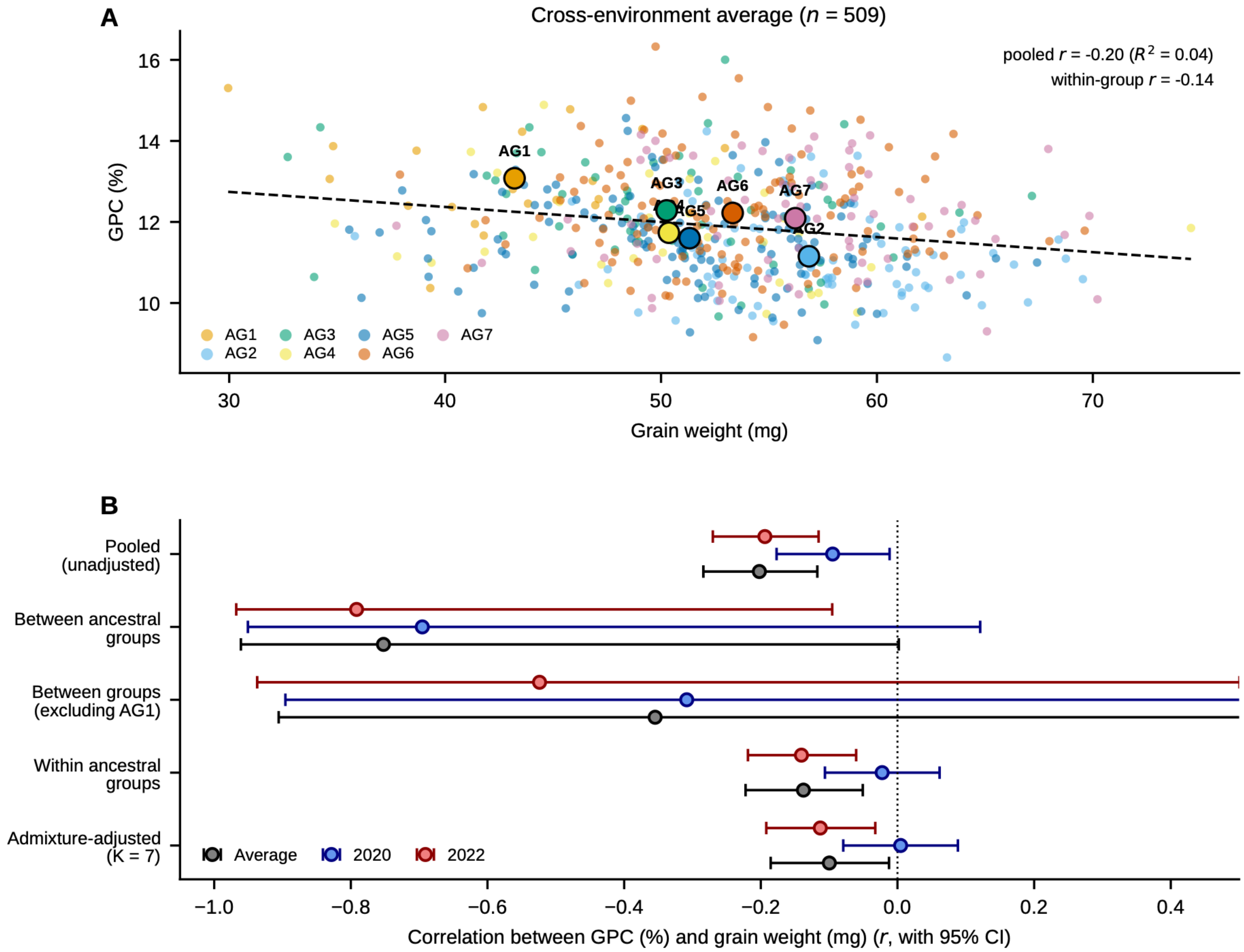

Fig. S5 Effect of population structure on the relationship between grain protein content and grain weight. Analyses use the same dataset as Fig. 3 (cross-environment average of genotypes phenotyped in 2020 and 2022). **(A)** GPC (%) against grain weight (mg), with genotypes coloured by ancestral group (AG1–AG7; **Cheng et al. 2024**), labelled outlined symbols showing ancestral group means, and the pooled regression shown as a dashed line. **(B)** Correlation between GPC and grain weight estimated as the pooled correlation, the correlation between ancestral group means with and without AG1, the pooled within-group correlation (ancestral group fitted as a fixed effect), and after continuous adjustment for K = 7 admixture proportions. Points show *r* with 95% confidence intervals for the cross-environment average and for each environment separately.

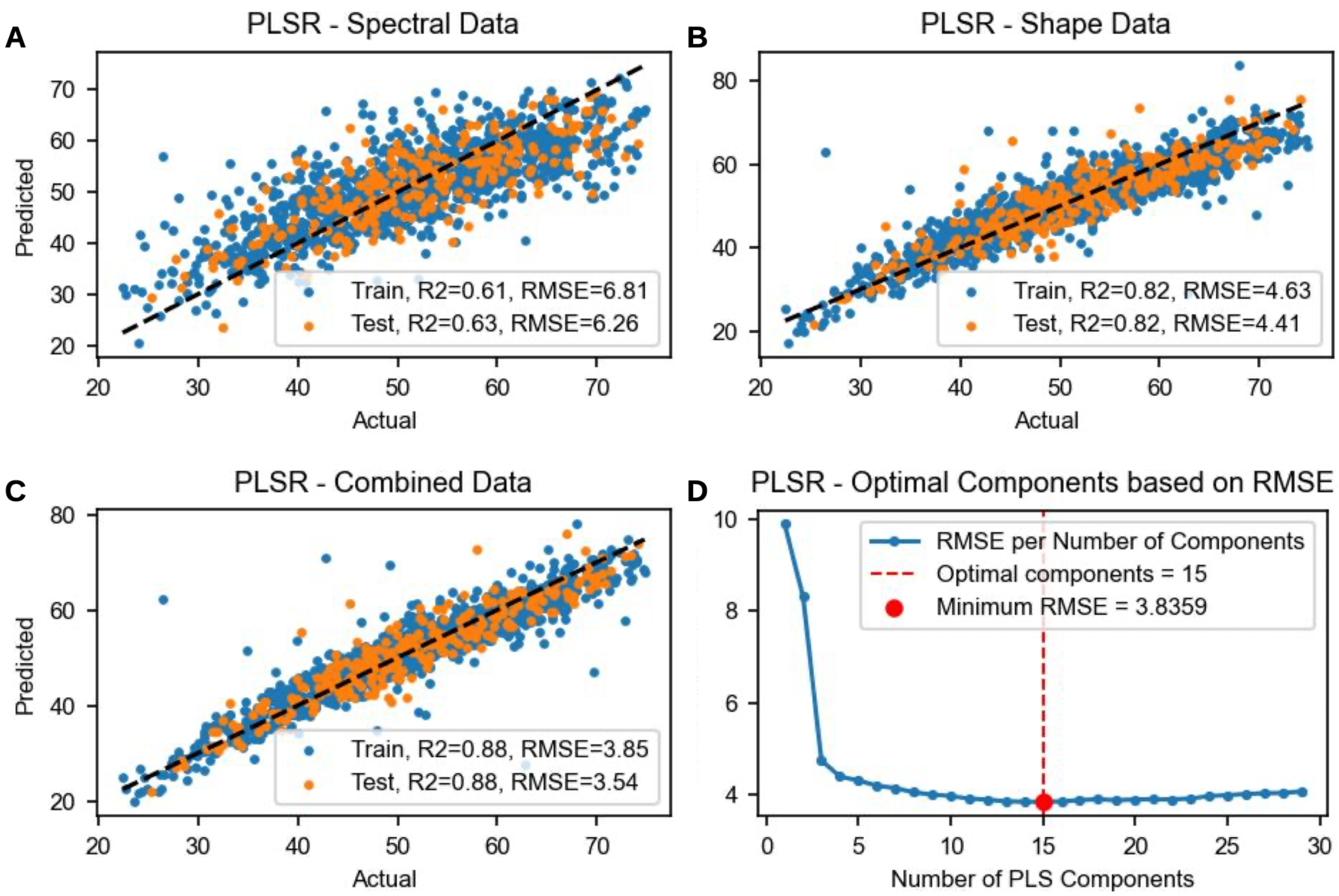

Fig. S6 Training a PLSR model for predicting grain weight. (A) Model trained using spectral features from HSI data. **(B)** Model trained using grain shape and size features that were calculated from HSI data dimensions. **(C)** Model trained using combined spectral and shape features. **(D)** Optimum number of components calculated using minimum RMSE method. Both spectral based and shape/size-based models showed equivalent fit to previously reported results for grain weight calibrations. Combining these features provided a more robust calibration model with RMSE below 4 mg grain weight.

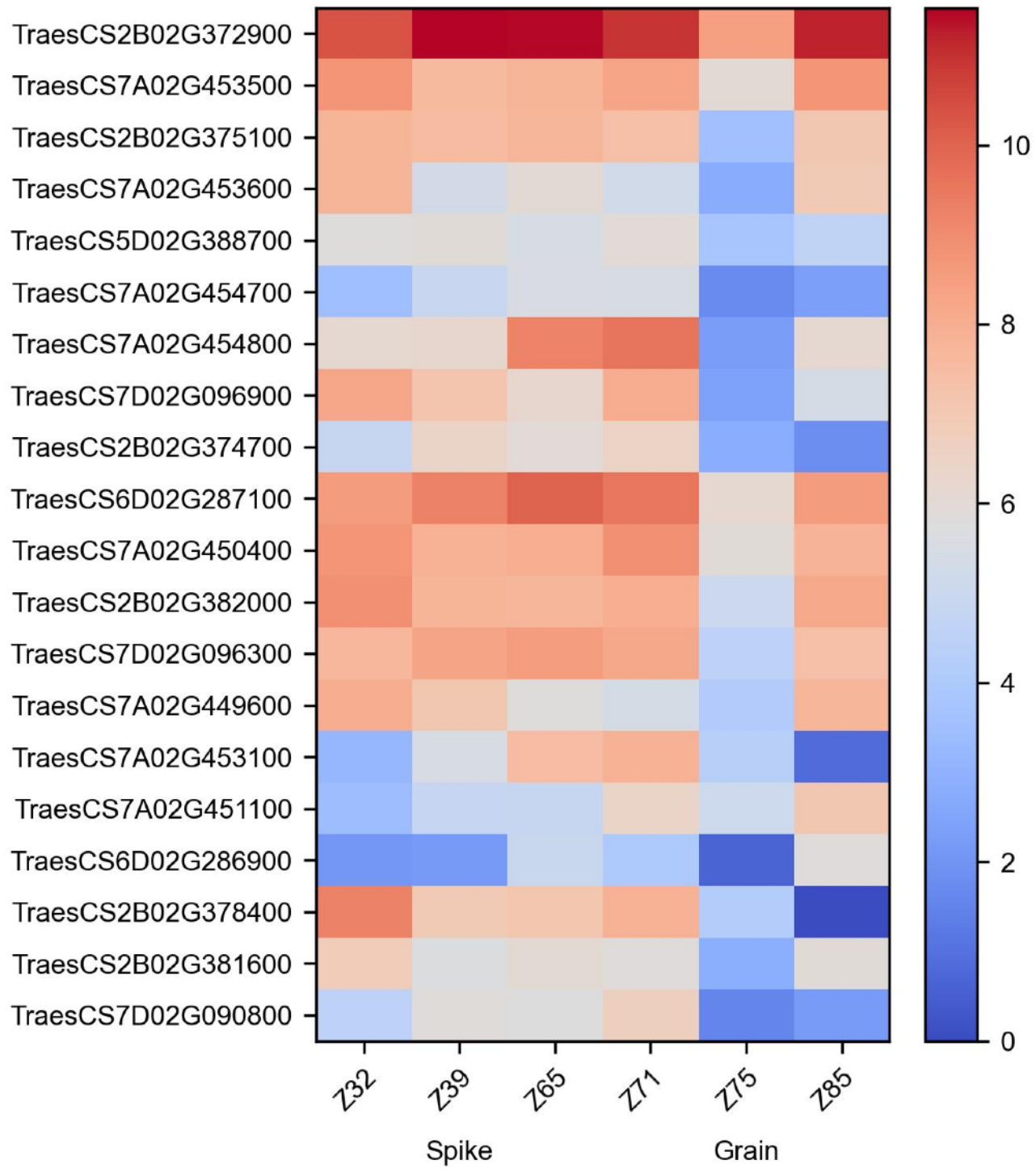

Fig. S7 Genes at six prioritised GPC loci expressed highly in both developing wheat spike and grain. Gene expressions were analysed from the developing wheat spike at Z32, Z39 and Z65, and grain at Z71, Z75 and Z85. These data are presented from previously published materials available at Wheat Expression Atlas and the developing stages correspond to timepoints critical for nitrogen uptake and remobilisation to grain. The expression values are normalized for the figure.

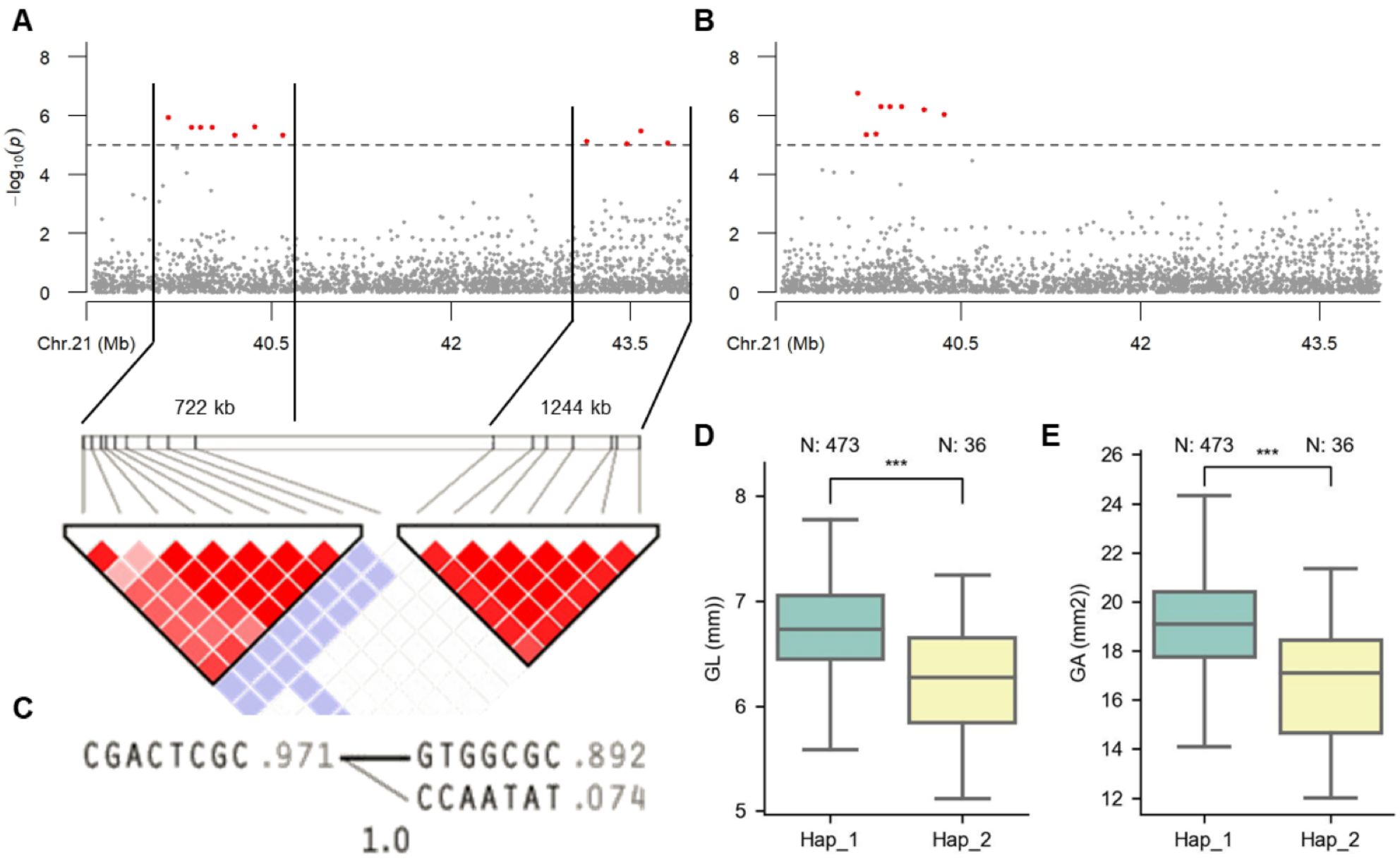

Fig. S8 Evaluation of GL and GA distribution in the Watkins collection at 7D locus. (A) Local Manhattan plot for GL shows significant SNPs forming two blocks. **(B)** Local Manhattan plot for GA shows only the presence of Block_1. **(C)** The block analysis shows two blocks to be significantly linked with Block_2 forming two haplotypes in 89.2% and 7.4% frequency. **(D)** and **(E)** Hap_2 significantly decrease GL and GA. Hap_2 was also associated with a decrease in GW and increase in GPC. Comparison of haplotypes was analysed using Independent Samples *t*-test. Asterisks show significance at *p* value < 0.05*, <0.01** and <0.001*** at alpha 0.05 for significance threshold.

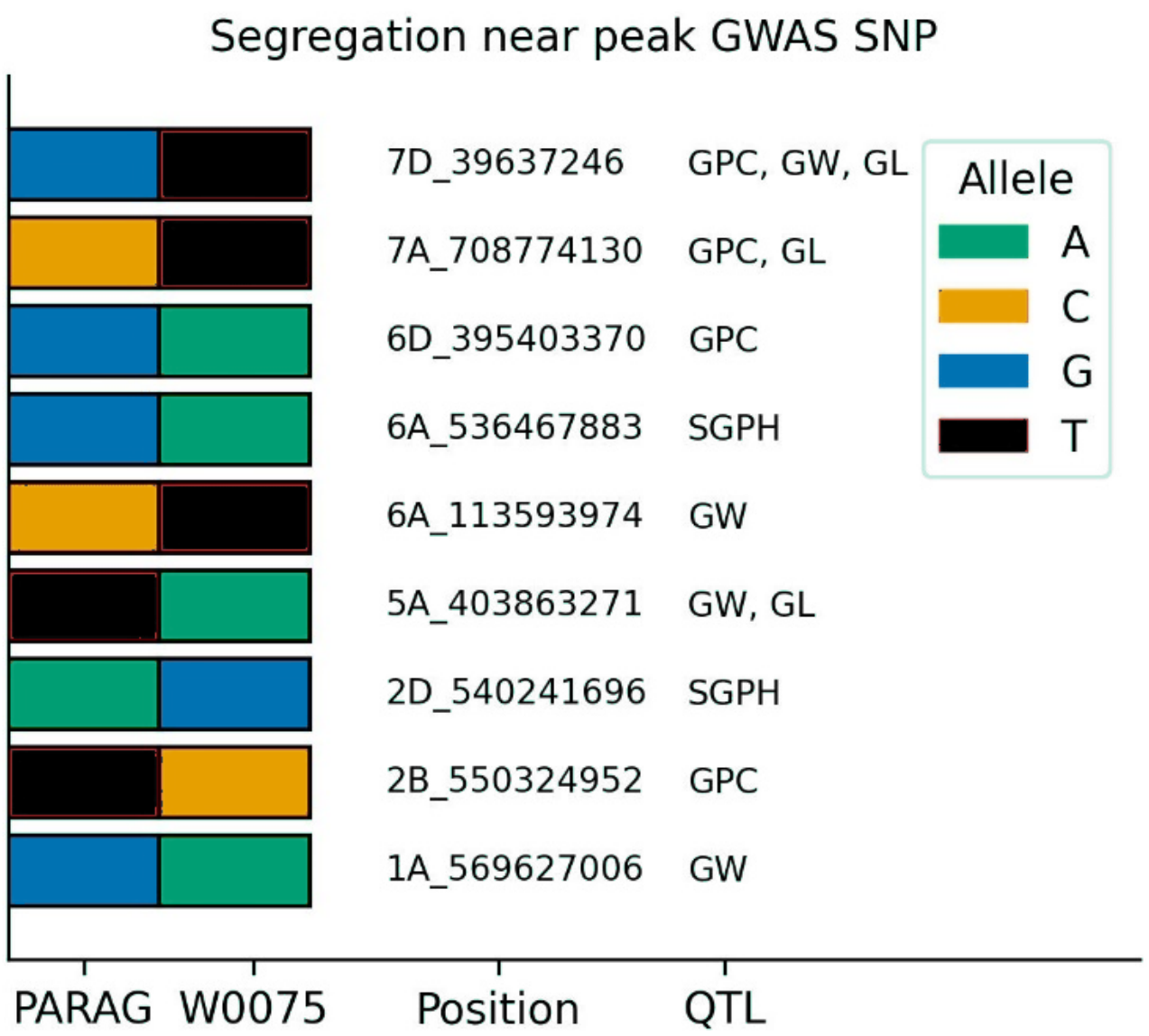

Fig. S9 SNP level variation between Paragon and WATDE0075. Using the whole-genome variants, 10 SNPs upstream and downstream from the peak GWAS SNP were checked. Position shows the nearest (from tag SNP) physical position where the two parents segregate. QTL column shows traits for which the loci were identified by GWAS.

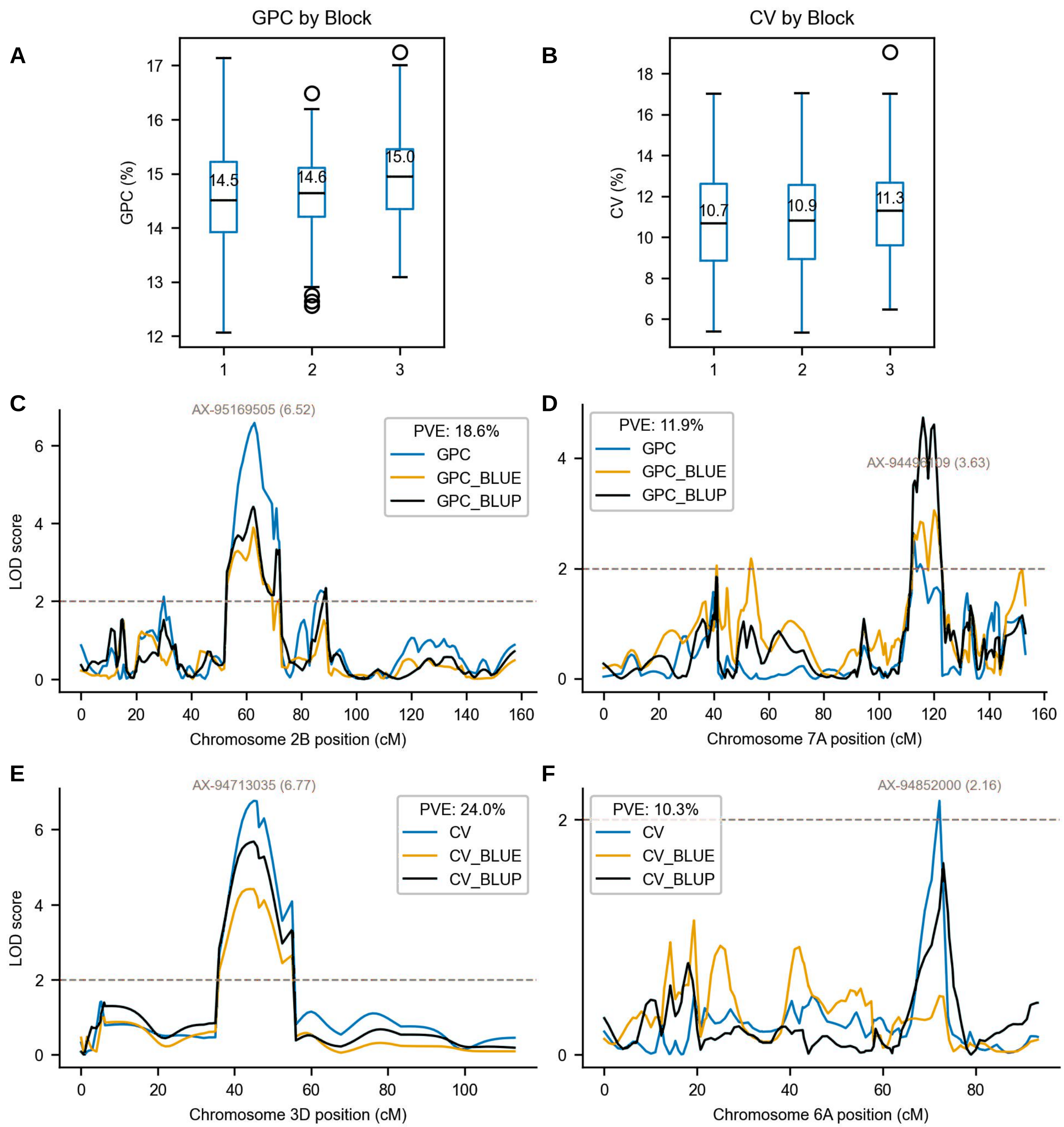

Fig. S10 QTL analysis for grain protein content and heterogeneity (CV%) in Paragon × WATDE0075. (A, B) GPC and CV% showed consistent spread across the three replicated blocks. **(C, D)** QTL identified for GPC on 2B and 7A wheat chromosomes explained 18.6% and 11.9% phenotypic variance for GPC and overlapped with GWAS loci. **(E, F)** QTL identified for heterogeneity in GPC on 3D (novel locus) and 6A (overlapping GWAS results) chromosomes explained 24.0% and 10.3% of the phenotypic variance.

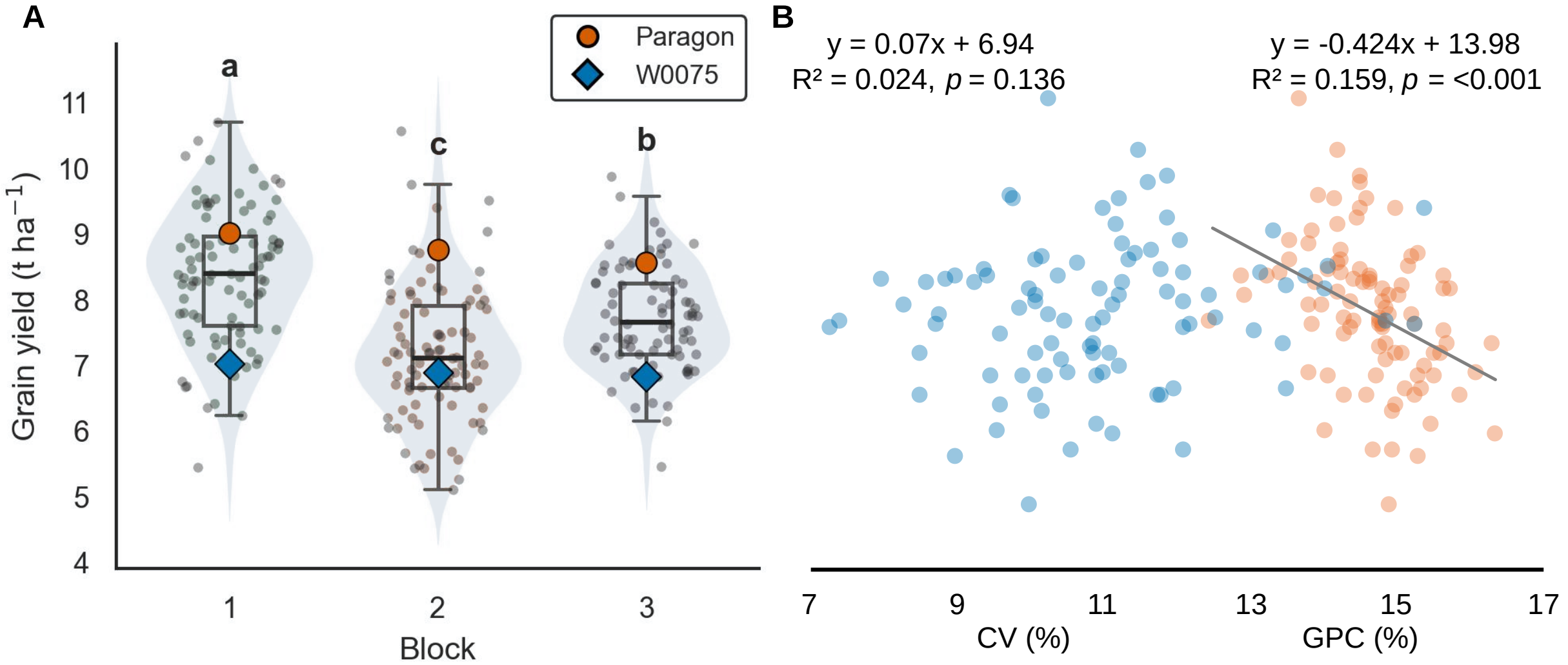

Fig. S11 Analysis of grain yield and its relationship with grain protein content (GPC%) and its heterogeneity (CV%) in the Sutton Bonington field experiment. (A) One-way ANOVA followed by Tukey's HSD for grain yield across field blocks shows a significant difference between yield across three blocks (n = 91). **(B)** Linear regression between grain yield and GPC heterogeneity (CV%) and mean-GPC. Grain yield (t/ha) shows no correlation with CV% while a significant negative correlation with GPC. For every 1% increase in GPC, grain yield decreases by 0.42 t/ha; however, a low R-squared (0.16) indicates that GPC only captured approximately 16% variation in grain yield.

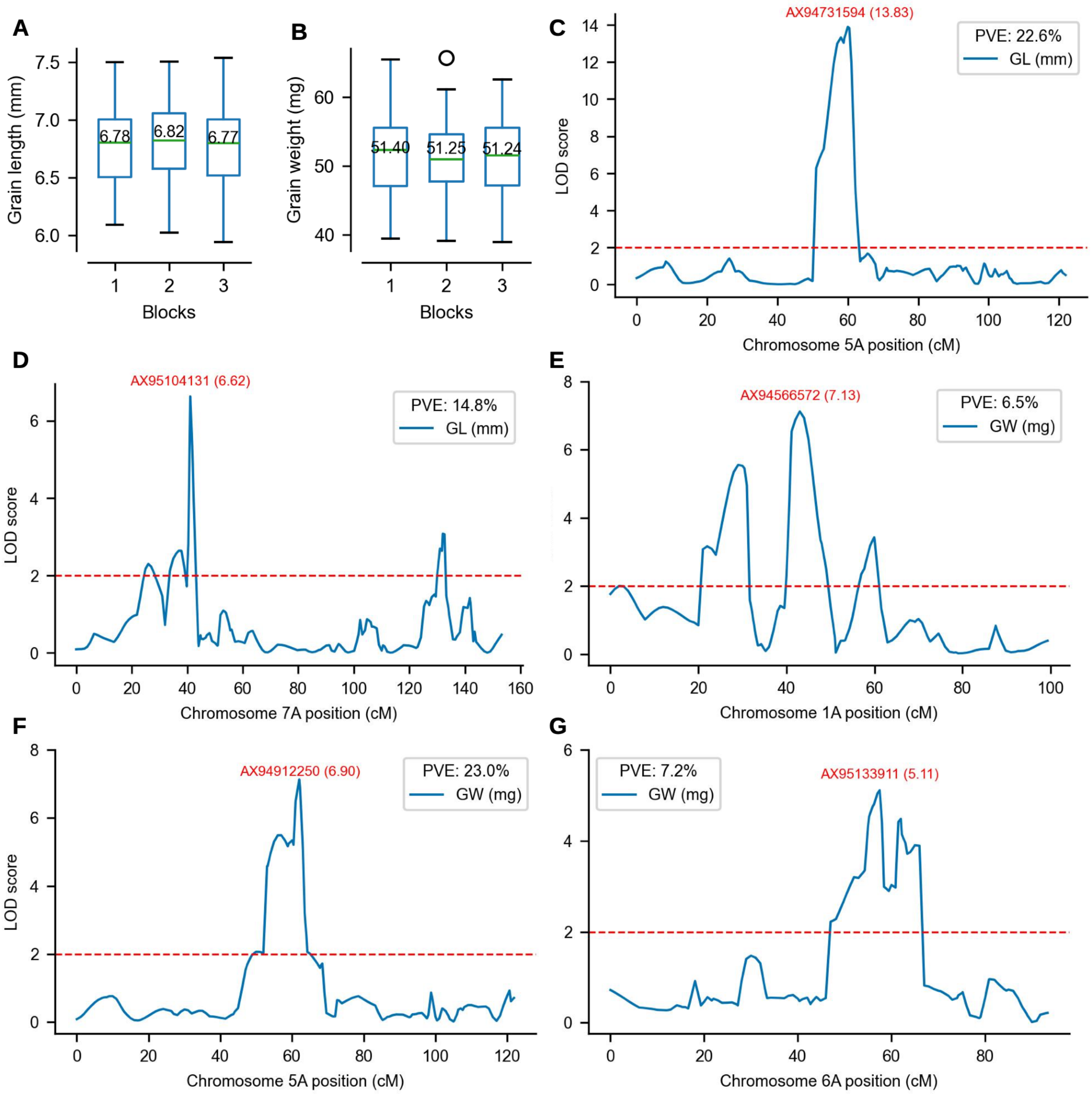

Fig. S12 QTL analysis for grain length and weight in Paragon × WATDE0075. (A, B) Phenotypic data by block shows both traits had a similar spread and comparable mean values across replicated blocks. **(C, D)** QTL identified on chromosomes 5A and 7A wheat chromosomes explain 22.6% and 14.6% phenotypic variation in grain length, respectively. **(E–G)** QTL identified for grain weight on 1A, 5A (same as grain length) and 6A chromosomes explain 6.5%, 23% and 7.2% phenotypic variation in grain weight. These QTL coincide with our GWAS peaks identified in the Watkins collection.

Table S1 Performance of different PLSR models on grain orientation specific test sets.

Table S2 GPC data from two field trials predicted by HSI. Only common genotypes across trials are presented.

Table S3 Loci associated with GPC in the Watkins wheat collection.

Table S4 Loci associated with GPC in the Watkins collection consistently across multiple datasets, along with their corresponding QTL from Cheng et al. (2024).

Table S5 QTL overlapping with GPC loci in bi-parental mapping populations reported by Cheng et al., (2024).

Table S6 QTL overlapping with GPC loci in GWAS panel reported by Cheng et al., (2024).

Table S7 QTL overlapping with GPC loci in NAM-GWAS panel reported by Cheng et al., (2024).

Table S8 Multi–environment loci associated with GPC in the Watkins collection.

Table S9 QTL reported for GPC in wheat from 1997–2021.

Table S10 Analysis of haplotypes from the Watkins HapMap overlapping with GPC loci identified in the present study.

Table S11 Functional annotation of genes at prioritised GPC loci with over 2-fold expression in grain and spike compared to stem and leaf.

Table S12 Watkins-unique variant genes expressed highly at prioritised GPC loci.

Table S13 Loci associated with grain weight in the Watkins collection.

Table S14 Loci associated with grain length and area in the Watkins collection.

Table S15 Effects of minor haplotypes at all loci tested, estimated separately in each environment, and tests of haplotype × environment interaction.

Table S16 QTL identified in Paragon × WATDE0075 mapping population.

Supplementary tables are Excel files which cannot be fitted here and are submitted separately.
